# Reputation or peer review? The role of outliers

**DOI:** 10.1007/s11192-018-2826-3

**Published:** 2018-07-09

**Authors:** Francisco Grimaldo, Mario Paolucci, Jordi Sabater-Mir

**Affiliations:** 1 Departament d’Informàtica, Universitat de València, Av. de la Universitat s/n, 46100 Burjassot, Spain; 2 Institute of Cognitive Sciences and Technologies, Italian National Research Council, Via Palestro 32, 00185 Rome, Italy; 3 Artificial Intelligence Research Institute, Spanish National Research Council, Campus UAB, 08193 Bellaterra, Spain

**Keywords:** Peer review, Reputation, Agent-based simulation, Multi-disciplinary science, Outliers, Information filter

## Abstract

We present an agent-based model of paper publication and consumption that allows to study the effect of two different evaluation mechanisms, peer review and reputation, on the quality of the manuscripts accessed by a scientific community. The model was empirically calibrated on two data sets, mono- and multi-disciplinary. Our results point out that disciplinary settings differ in the rapidity with which they deal with extreme events—papers that have an extremely high quality, that we call outliers. In the mono-disciplinary case, reputation is better than traditional peer review to optimize the quality of papers read by researchers. In the multi-disciplinary case, if the quality landscape is relatively flat, a reputation system also performs better. In the presence of outliers, peer review is more effective. Our simulation suggests that a reputation system could perform better than peer review as a scientific information filter for quality except when research is multi-disciplinary and in a field where outliers exist.

**Electronic supplementary material:**

The online version of this article (10.1007/s11192-018-2826-3) contains supplementary material, which is available to authorized users.

## Introduction

Research, as a social process aimed to the discovery of natural and social truth, has been ever growing since the adoption of the scientific method. It has been a growth in numbers: scientists, subject, papers (doubling every 9 years Bornmann and Mutz 2015); an increase in public and private funds (in absolute, although maybe not in relative terms); and also an intensification of its effects on the everyday life (De Solla Price 1963).

An ecosystem on its own, the research social system evolved and reacted to its growth by adapting its practices and by developing new ones such as peer review. If a researcher could be blissfully unaware of its existence at the beginning of the last century, peer review in the form we know it today became common knowledge after the second world war. There was a very simple reason for that: editors could not sustain alone the needs of journal publishing, in time and in field coverage.

In recent times, the combined effects of growth and technological changes put the research system, and peer review especially, at a crossroad. Should the research publication system stay on its path, or should it take a new direction, getting rid of peer review to embrace instead reputation systems (Pinyol and Sabater-Mir 2013)? Reputation systems (Dellarocas 2010) are the unsung heroes of the social webs, helping to sort out information overload and to build trust. With current technology, it is easy to imagine a system in which papers do not get subject to peer review, but are published immediately and then scored online with a reputation system comparable, for example, to the one in eBay. Papers with higher scores will attract more reads. How would that compare to peer review?

The interest of this comparison is manifold. First, one could argue that reputed referees should not be substituted by non-experts. However, it is a fact that the growth in the number of submissions threatens the sustainability of a system that leans on voluntary cooperation (Lortie 2011; Kovanis et al. 2016b). A reputation system based on the opinions of scientists after reading state-of-the-art articles may help reduce reviewing workload, an effort that could be dedicated to the production of new science. Second, drafts need to be available in order to be read and assessed but on-line repositories are more and more used and solve any physical publication space limitation. Third, the citation and reputation systems can conceptually overlap as both recognize quality but the first one can be biased towards the aggregate reputation of the journal and might develop more slowly due to the delay introduced by the publication time. Last but not least, reputation might be stronger as a drive to decide what to read since paper level scores could better match the heterogeneous quality of manuscripts published in a journal and result in a fairer visibility.

Here, we provide an agent-based simulation to help understanding the process of science generation and perusal, aiming to measure information flow as mediated either by peer review or by reputation. In the rest of this paper, we will show how we grounded our system on stylized facts, how we built and calibrated our agent-based simulation, and we will examine results showing the role of exceptional papers in discriminating peer review from reputation for what concerns the quality of read papers.

## State of the art

Science production and evaluation has been profusely modelled in the past with the aim of understanding and reproducing the mechanisms that underlie what has been known as the social processes of science (Meyer 2011; Edmonds et al. 2011). This topic includes aspects such as paper generation, co-authorship, judgment, selection, publication or citation, just to name a few. The state-of-the-art existing models mainly differ in the elements considered, their dynamic features and the stylized facts they reproduce. In the following, we will highlight some relevant characteristics of a non-exhaustive list of simulation approaches that tackle how scientists produce papers and evaluate them.

Table 1 shows the main details about a selection of models that have been proposed over the last 20 years and allows us to compare them with our approach. The early models (Gilbert 1997; Saam and Reiter 1999; Börner et al. 2004; Sun and Naveh 2009) are mostly concerned with science production and, thus, focus on reproducing the left-skewed distributions found in reality for paper production and citation. The entities represented in these models usually consist of authors and papers. Besides knowledge generation, they deal with co-authorship, manuscript reading and paper citation mechanisms.

**Table 1:** Main characteristics of state-of-the-art models of science production and evaluation

	Gilbert (1997)	Saam and Reiter (1999)	Börner et al. (2004)	Sun and Naveh (2009)	Osman et al. (2012)	Allesina (2012)	Squazzoni and Gandelli (2013) and Cabotà et al. (2014)	Grimaldo and Paolucci (2013) and Paolucci and Grimaldo (2014)	Gu et al. (2015)	Kovanis et al. (2016a, 2017)	Righi and Takács (2017)	This paper’s approach
*Entities included*												
Authors	$\checkmark$	$\checkmark$	$\checkmark$	$\checkmark$	$\checkmark$	$\checkmark$	$\checkmark$	$\checkmark$	$\checkmark$	$\checkmark$	$\checkmark$	$\checkmark$
Reviewers					$\checkmark$	$\checkmark$	$\checkmark$	$\checkmark$		$\checkmark$	$\checkmark$	$\checkmark$
Editors					$\checkmark$	$\checkmark$	$\checkmark$	$\checkmark$		$\checkmark$	$\checkmark$	$\checkmark$
Papers	$\checkmark$		$\checkmark$	$\checkmark$	$\checkmark$	$\checkmark$	$\checkmark$	$\checkmark$		$\checkmark$	$\checkmark$	$\checkmark$
Reviews							$\checkmark$	$\checkmark$		$\checkmark$	$\checkmark$	$\checkmark$
Journals					$\checkmark$	$\checkmark$	$\checkmark$	$\checkmark$	$\checkmark$	$\checkmark$		$\checkmark$
*Model features*												
Knowledge generation	$\checkmark$			$\checkmark$								
Co-authorship	$\checkmark$		$\checkmark$		$\checkmark$							$\checkmark$
Submission strategy					$\checkmark$	$\checkmark$			$\checkmark$	$\checkmark$		$\checkmark$
Peer review process					$\checkmark$	$\checkmark$	$\checkmark$	$\checkmark$		$\checkmark$	$\checkmark$	$\checkmark$
Reviewer selection					$\checkmark$		$\checkmark$	$\checkmark$			$\checkmark$	
Citation mechanism	$\checkmark$	$\checkmark$	$\checkmark$									
Paper reading			$\checkmark$							$\checkmark$		$\checkmark$
Strategic behaviour							$\checkmark$	$\checkmark$	$\checkmark$	$\checkmark$	$\checkmark$	
*Measures considered*												
Reputation		$\checkmark$			$\checkmark$						$\checkmark$	$\checkmark$
Quality		$\checkmark$		$\checkmark$	$\checkmark$	$\checkmark$	$\checkmark$	$\checkmark$		$\checkmark$	$\checkmark$	$\checkmark$
Resources							$\checkmark$			$\checkmark$		
Production distribution	$\checkmark$	$\checkmark$	$\checkmark$	$\checkmark$		$\checkmark$			$\checkmark$	$\checkmark$		$\checkmark$
Citation distribution	$\checkmark$	$\checkmark$	$\checkmark$									

Reviewers, reviews, editors and journals have also been represented in the models in Table 1. These approaches have normally addressed the behavioral side of the process and the effect of different evaluation policies on the quality of the research being published. Strategic behavior of scientists when submitting papers can tilt the balance for or against productivity (Gu et al. 2015) but reputation mechanisms can be applied to encourage ‘good’ research behavior (Osman et al. 2012) and promote the investment of time for reviewing papers (Righi and Takács 2017). Scientific competition for scarce resources may lead to the emergence of reciprocal and cheating reviewing codes of conduct (Squazzoni and Gandelli 2013; Paolucci and Grimaldo 2014), whereas reviewer selection editorial policies (Grimaldo and Paolucci 2013; Cabotà et al. 2014) can help keep these biases under control. Last but not least, variants of the peer review process itself have been analyzed in search for alternative protocols that boost the efficiency of the system, e.g., by having journals bidding for papers (Allesina 2012) or cascading the reviews of rejected manuscripts (Kovanis et al. 2016a, 2017).

## Research question

The core research question in this paper is: how does the traditional peer review system compare to a distributed, crowd-sourced evaluation, and in particular to an evaluation based on reputation? Obviously, a large-scale, automated, distributed reputation system was not available when peer review became commonplace. Science reviewing, however, started long before peer review—in fact, peer review did not exist in the form we know it until after the war (Biagioli 2002)—in the form of correspondence between scientists. Personal correspondence followed personal connections, which could be considered already a form of reputation-based networking.

Nowadays, the prevalence of peer review is challenged. Anecdotal failures (some of then engineered) are reported in high-diffusion journals; these express, more than an effectively accurate analysis of the phenomenon, a diffused dissatisfaction of researchers with the process (Tennant et al. 2017). We might be witnessing the replacement of peer review with new mechanisms—reputation being our example.

While the issue has already been discussed (Frishauf 2009), we think that an answer to this research question should be obtained from a computational approach, also taking into account time and progressive knowledge accumulation. We have thus developed a computational model of science production and consumption to answer the question: how do different quality distributions influence the consumption of science under different filtering mechanisms—specifically, peer review and reputation? To answer this question, we introduce our model, focusing on different quality distributions and on the two mechanisms of reputation and peer reviews as information filters.

## The model

### One model of production, two sub-models of readership

In order to compare the two filters-reputation and peer review—we have built a simulation model^1^ that includes a common paper production mechanism and then runs two distinct sub-models for accessing papers: one driven by reputation and the other by peer review.

The simulation cycle of our model begins with paper production, generating the papers for the current time step and adding them to the papers pool. Afterwards, we apply either a simulated peer review process or a simulated reputation mechanism, both driving parallel reading processes. In the former case, scientists read papers as prioritized by journal’s quality whereas, in the latter case, they read papers as prioritized by their reputation.

Thus, the two sub-models share the same ground truth for what regards paper and scientist quality. This guarantees a clean comparison, made with exactly the same papers in the system, at the cost of ignoring the feedback from reading towards paper production.

### Sources of randomness

All models of science, including models of peer review as the current one, must deal with the problem of paper quality. In this paper, we choose the approach of generating quality from an aleatory distribution. This entails a choice for that distribution; in most applicable definitions, quality is path-dependent, including at the very least both veracity (a paper without mistakes is a good paper) and novelty (a clean derivation of an existing result will not have the same impact as the first derivation of the same). It could be argued that impact should also be considered—although impact can only be meaningfully measured *a posteriori*, adding even more intricate path dependence to the picture. With all those factors to consider, the question to be asked is what are the measurable indicators of this inaccessible quality.

Most models of science use citations as a proxy for quality, estimating the cause by the effect. While this is also an oversimplification, we are going to adopt it for the purposes of the present work. However, although there is a consensus on the distribution of citations being right skewed, with many papers collecting little citations and few papers collecting many citations each, the fit on empirical data is never perfect. Different fields, different journals, different time scales can give rise to different fits. This inspired us to apply two different distributions to our simulation. These are the power law and the exponential. We apply these two distributions to extract the quality of papers, the initial reputation of the scientists and the quality of the journals.

Long tail distributions have several interpretations, generally including some form of selected positive feedback or (the positive part of) Matthew effect. Here, we focus on their ability to describe large groups with substantial inequality, where a few individuals exhibit, for some measure, values exceeding the sum of every other individual. We know this is true for usual measures of science: top authors cite each other and have access to fast track reviews (Sarigol et al. 2017). In economics, there is a strong correlation between the ranking of an author’s first employer and the number of citations received (Baghestanian and Popov 2014), making it the most important predictor for early career success. These individuals, in our case being innovative papers of exceptional quality, will be called *outliers* in the rest of the paper.

### Elements of the model

**Fig. 1:**
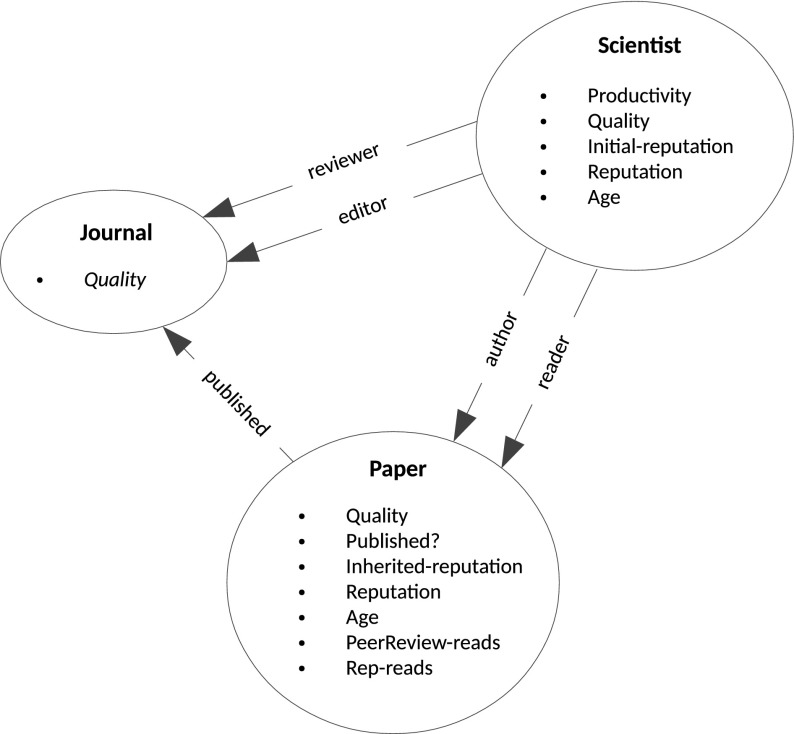
Main entities of the simulation, their properties and relationships

**Fig. 2:**
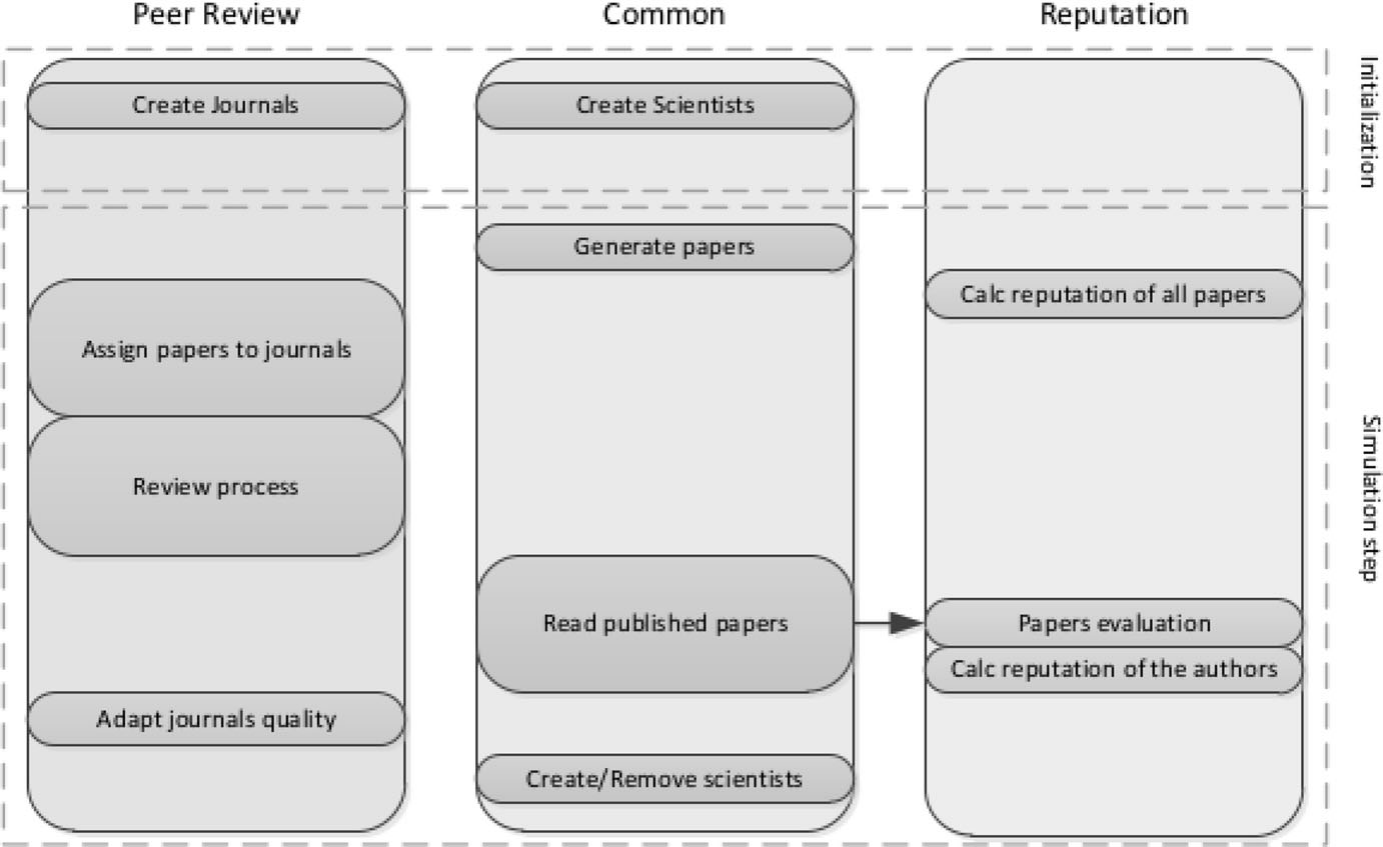
Execution flow, from top to bottom, in three lanes. The common lane provides the two information filters with the same ground truth

Following the approach used by other paper production models (see Payette 2012 for a review), we consider as main entities the papers, the journals and the scientists, that play the roles of authors, reviewers and editors. Figure 1 illustrates the relation between these entities and the properties that characterize each one of them. Figure 2 shows the execution flow of the simulation. Some stages are common to both the peer review and the reputation sub-models, while others are specific.

At the initialization stage, the simulation creates the initial set of scientists. A scientist (as shown in Fig. 1) is characterized by several state variables that are initialized when it is created. We list them below.

- *Productivity* Measures the number of papers that the scientist can produce every simulation step as well as the number of papers that can read. The productivity is initialized with an exponentially distributed random number that ranges from 1 to a customizable parameter.
- *Quality* Nominal quality of the scientist. We use random generators of positive quality following either a *Zipf* or *exponential* distribution.
- *Initial reputation* Reputation value assigned initially to the scientist. Uses the same random generator of *quality* parameter.
- *Reputation* Current reputation of the scientist. This value takes the *initial reputation* as starting value and changes when the scientist starts to co-author papers and these papers are read.
- *Age* The “scientific age” of the scientist. Starts from 1 increases with simulation steps.

Journals, on initialisation, are assigned an editor at random and a customizable number of reviewers from the initial set of scientists. Journal qualities are calculated by creating a quality sample from the same distribution used for initializing scientists (i.e. *Zipf* or *exponential*) and then placing journals so as to separate the sample in bins of equal size. This procedure allows the placement of journals in a qualitatively comparable way, independent of the specific distribution chosen. After creating the scientists and the journals, the simulation cycle continues with the paper generation stage.

### Paper generation

Paper production is simulated as the main activity of scientists. In our model, a group of scientists is put together to produce a paper in simulated co-authorship. The sequence for the generation of papers is the following:

- *Create drafts* The number of drafts to be created corresponds to the total productivity divided by the average authors per paper. Draft qualities are at this point independent of author qualities and they are calculated as a random sample of the *Zipf* or *exponential* distributions used to generate scientist qualities. The initial quality of a draft represents the quality of the “idea” presented in that manuscript.
- *Assign co-authors to drafts* The first author is assigned randomly from those scientists with free production capacity. Then, the co-authors can be selected randomly or selected from the pool of authors that had previously written a paper with one of the already assigned authors.
- *Calculate the quality of the contribution* Once the paper is produced, its final quality is calculated as the average between the quality of its co-authors and the quality of the “idea” in the paper that was randomly generated as detailed above.

Once the quality of the paper is attributed, the authors’ quality is modified as if attracted towards the paper’s value, weighted by an adaptation rate parameters (defaulting at 0.1). In other words, working with better scientists or having a great idea improves a scientist’ quality. The opposite is also true.

### Peer review sub-model

In the peer review sub-model, after the paper generation stage there is the submission and review stage. The manuscripts just created are submitted to journals so they can be reviewed. The submission is made by one of the co-authors (selected randomly) who chooses a journal that has a quality level similar to the paper’s quality, with a noise extracted from the distribution used for the quality.

Once a draft is submitted, the review process starts with the following steps:

- Select randomly a set of reviewers from those associated to the journal.
- Ask each reviewer to evaluate the paper. The reviewer accepts the paper for publication if the quality of the paper is higher than the quality of the journal +/- a noise that is inversely proportional to the nominal quality of the scientist that is acting as a reviewer.^2^ In other words, the capacity of the reviewer determines the level of noise in the acceptance of a paper.
- The reviewers say “yes” or “no”. If the number of “yes” is higher then the editors makes the decision to accept the paper, if not it is rejected.

After the reading stage (see Sect. 4.6), journals update their quality on the base of the new quality distribution of accepted papers. The new journal quality is calculated as the one that would divide the current published set in equal parts. This simulates an adaptive process on the part of journals with the purpose of maintaining a clearly separate identity.

### The reading stage

The reading stage is common for both the peer review and the reputation sub-model. This stage simulates the process of reading papers available to the scientific community. In the case of peer review, this means the papers that have been accepted for publication in a journal. In the case of reputation, this means all papers that have been generated till now. A scientist can read the same paper more than once.

In a simulation step, each scientist can read a number of papers that is proportional to its productivity. Therefore, being capable of reading only a limited amount of papers, scientist must choose. The choice is driven by different mechanisms in the two compared models. In the peer review model, the chances of a paper to be read depends on the quality of the journal where it has been published and from the age of the paper while in the reputation model it depends on its current reputation score and age. In both cases the system uses a weighted lottery over the candidate papers.

The visibility that a paper has according to its age is modeled following Parolo et al. (2015) that states that during the first 3 years, the visibility of a paper increases till its maximum and after then, it decreases exponentially.

### Reputation sub-model

After paper generation, the reputation sub-model calculates what we call the *inherited reputation*. The *inherited reputation* of a paper is the mean of the reputation of the authors. Then, the sub-model calculates the current *reputation* of that paper.

The *reputation* combines the *inherited reputation* value with the mean of the evaluations that the paper has received till then. If there are no evaluations yet, the *reputation* is equal to the *inherited reputation*. If the number of evaluations is higher than a predefined threshold, only the evaluations are used to calculate the *reputation* and the *inherited reputation* is neglected. Otherwise, a weighted mean between evaluations and *inherited reputation* is employed. The idea behind this mechanism is that while there are not evaluations of the paper or there are few of them, the reputation of the authors determines the reputation of the paper. Once there are enough evaluations, the authors’ previous reputation does not enter the calculation anymore.

In the reputation scenario, papers do not need to get through the gates of peer review. Every paper written is immediately accessible for reading. A scientist, after reading a paper, is asked to evaluate it. This evaluation takes as a basis the real quality of the paper and adds a random noise (with the same formula as for the peer review evaluation) that is a function of the quality of the evaluator. On the one hand, a scientist with a very low quality will generate evaluations that can differ a lot from the real quality of the paper. On the other hand, a scientist with a high quality will generate evaluations that are very close to the real quality of the paper. Papers keep the list of their evaluation scores, as in post-publication peer review scenarios.

Finally, the reputation of the scientists is updated. The reputation of a scientist is calculated in a similar way than the reputation of a paper. The *reputation* combines the *initial reputation* value with the mean of the reputations of the papers that has co-authored (other aggregation methods, possibly focusing on brilliant or recent papers, remain future work). If the scientist has not co-authored a paper yet, the *reputation* is equal to the *initial reputation*. If the number of paper co-authored is higher than a threshold only the reputation of the papers is used to calculate the *reputation* and the *initial reputation* is neglected. Otherwise, the number of co-authored papers determine the weight that the reputation of those papers have in front of the *initial reputation*.

## Calibration

Our simulation model contains a large number of parameters that needs to be set. This has been done by looking at concrete cases, exploiting the Scopus^3^ database. We present here two different calibration exercises, starting with a mono-disciplinary journal, catering to the field of artificial agents and multi-agent systems. The second extraction we use for calibration is a term search. In a self-referential way, for this second set we have chosen to collect the multi-disciplinary publications dealing with peer review.

### Calibration on the mono-disciplinary set: the JAAMAS extraction

To perform the first calibration, we extracted from Scopus the set of all papers published up to the year 2016 in the Journal of Artificial Agents and Multi Agent Systems (JAAMAS),^4^ together with the number of citations they have collected to the date of extraction (March 31st, 2017). We only consider papers with at least one citation. A specialized journal, JAAMAS caters to the subset of the distributed artificial intelligence community that has an interest in agents. Table 2 summarizes this dataset quantitatively. We observe a set of authors with a low overall productivity (as confined to JAAMAS only), where most of the authors (about 800 over 1000) contribute just with one paper; the top author for productivity contributing with 14 papers, less than one per each of the 19 years in the set.

**Fig. 3:**
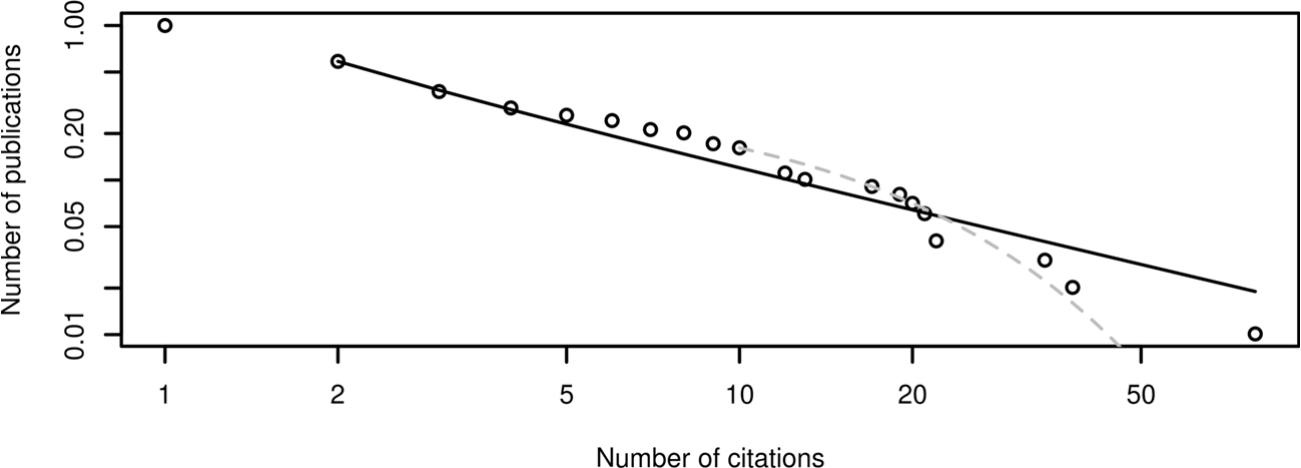
Log–log cumulative distribution of citations for the mono-disciplinary (JAAMAS) extraction. The lines show the power law fit (solid black, xmin = 2) and the exponential fit (dashed gray, xmin = 10)

Fitting a set of data with a distribution always leaves some arbitrariness, for example in the choice of target distribution. For the purposes of the current work, in accordance with the stylized facts found in the literature, we have bound our search only to the exponential and power law distribution, in their aspect of forbidding or allowing, respectively, out-of-scale top values (outliers) to appear. For what concerns the shape of citations vs. number of authors, the distribution recorded in the Scopus dataset for JAAMAS could be matched stepwise, first by a power law with a slope of 1.88, followed then by an exponential cutoff fitted with an exponential $\lambda$ value of 0.090. The fit is represented graphically in Fig. 3.

### Calibration on a multi-disciplinary extraction: the peer review extraction

To perform the second calibration, we queried Scopus about documents having ”peer review” as a keyword or as part of the paper title. As this term is rather frequent in scientific manuscripts, the resulting extraction needs to be deduplicated and cleaned up as follows.

First of all, we extracted the set of publications, considering only journals and not conference proceedings, book chapters or editorial notes. The resulting data represented the multiple and diverse disciplines that have studied peer review along time. It included 1528 journals, mostly publishing a very low number of papers in the period between 1993 and 2016. For instance, more than 200 of these journals had only one paper in the whole period. Then, we excluded journals with just a few papers in the dataset, considering them non-representative of the publishing scene about peer review. Otherwise, we would be calibrating our model on a scattered set with no underlying community, while our model aims to describe the publication results and the reading prioritization processes inside a well defined community. Thus, we removed from the set all the journals publishing less than 10 papers in the 44 years considered.

**Fig. 4:**
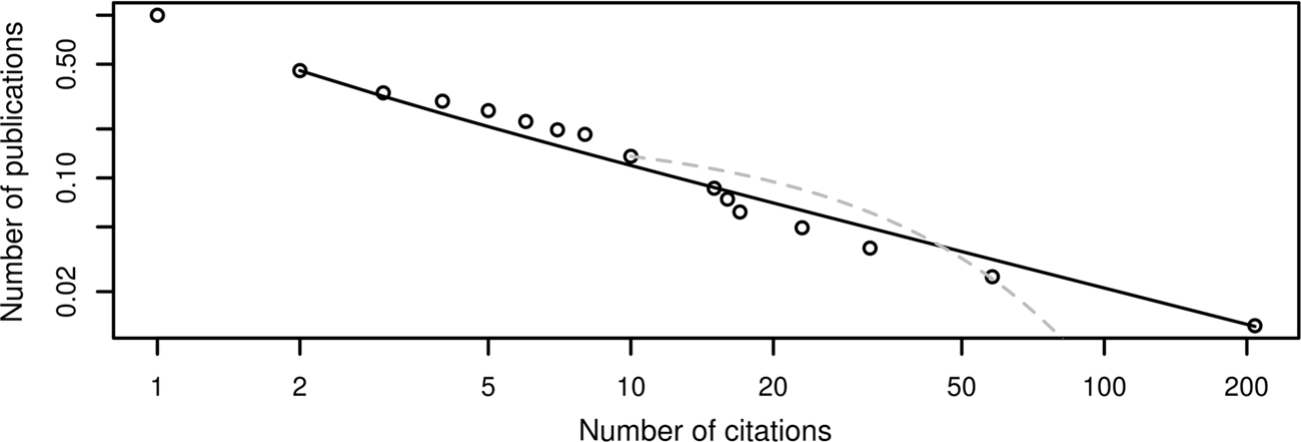
Log–log cumulative distribution of citations for the multi-disciplinary (peer review) extraction. The lines show the power law fit (solid black, xmin = 2) and the exponential fit (dashed gray, xmin = 10)

The resulting set is characterized numerically in Table 2. Like the previous one, this is also a set with a low overall productivity, as confined to just research about one topic, where most authors contribute just with one paper. The distribution of the citations recorded in the reduced data set follows an approximate power law with a slope of 1.72 (see Fig. 4). In this case, there is no visible exponential cutoff; an attempt at fitting data with an exponential produced a $\lambda$ value of 0.028, but the fit was so low that it could be discarded just by observation.

**Table 2:** Summary of the mono- and multi-disciplinary data extractions

Data overview	Mono-disciplinary	Multi-disciplinary
Years considered	1998–2016 (19 years)	1973–2016 (44 years)
Number of papers	516	520
Number of authors	1021	848
Number of sources	1	36
Average authors per paper	2.94	2.28
Publication rate per author	0.027	0.014
Power law fit (exponent, xmin)	(1.88, 2)	(1.72, 2)
Exponential fit (lambda, xmin)	(0.090, 10)	(0.028, 10)

## Simulation results

In order to address our research question introduced in Sect. 3, we have performed two sets of simulations calibrated with the results from the mono-disciplinary and the multi-disciplinary extractions above. In this way, we aim to show how peer review and reputation differ for the aggregate quality of reads, both with and without outliers. Results are summarized below.

### Results for the mono-disciplinary calibration

The first simulation run that we present is calibrated with the parameters obtained from the JAAMAS extraction, which concerns a set of papers and authors in a relatively homogeneous and coherent discipline. The adaptation of these parameters to our model is presented in Table 3 (left column). The productivity is calculated so as to have a final number of published papers (for the peer review sub-model; in the reputation one, all papers are considered accessible) comparable to the one in Table 3—that is, around 500 published papers with an acceptance rate of one half. On this base, we run two simulation scenarios with different quality distributions, with and without outliers—the first set following an exponential distribution, the second following a Zipf distribution.

**Table 3:** Model parameters for the simulations in the mono-disciplinary and multi-disciplinary calibrations. The main differences are the number of journals and the time period

Parameter	Mono-disciplinary	Multi-disciplinary
Years (steps)	19	44
Scientists	1000	848
Journals	1	36
Productivity (mean)	0.15	0.07
Reading multiplier	5	5
Authors per paper (mean)	2.95	2.28
Reviewers per paper	3	3
Adaptation rate	0.1	0.1
Zipf distribution (exponent)	1.87	1.72
Exponential distribution (mean)	0.09	0.09

**Fig. 5:**
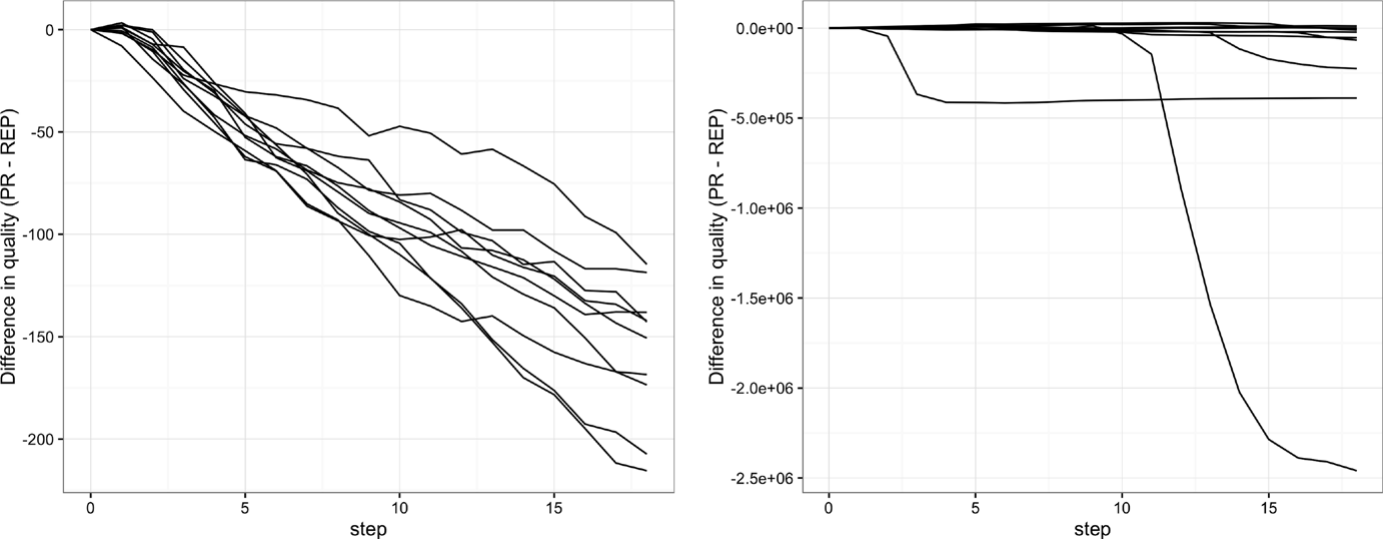
Results for the simulations calibrated using the JAAMAS data extraction with different quality distributions: exponential (left) and Zipf (right)

Figure 5 shows, for ten simulation runs, the difference in quality between the peer review and reputation sub-models under the same model of production (see Sect. 4.1). Negative values mean that reputation results in a higher amount of overall quality reads. This is the case, indeed, for both the exponential and the Zipf quality distributions. In the first scenario (left) the time evolution of this difference is smooth, approximately linear. In the Zipf case (right) the time evolution is characterized by sudden jumps, generated from the appearance of an outlier, in this case a paper of extraordinary quality that reputation promotes better than peer review. While—especially for the Zipf case—the actual values of the 10 repetitions are widely different, the common pattern here is that, for most of the runs, reputation results more efficient than peer review in terms of total quality read.

**Fig. 6:**
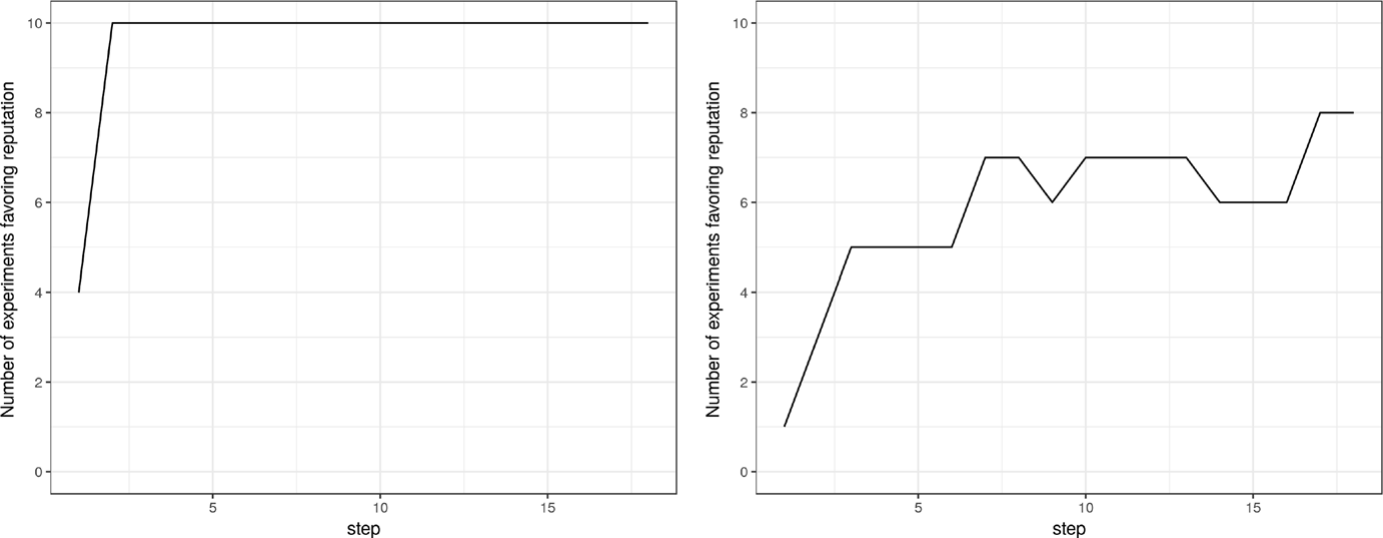
Number of simulations where reputation prevails in the mono-disciplinary case. In both cases, at the end of the period reputation is more efficient

This qualitative result is evident in Fig. 6, where we simply plot the number of simulations in which the reputation mechanism is more efficient against time—that is, the number of simulations with a quality difference under zero. In the exponential scenario, the prevalence of reputation is clear. In the Zipf scenario, reputation prevails too, but only after an initial phase in which both approaches seem to work equally well. It seems that, to have a clear advantage, reputation needs to wait for the emergence of the outliers, which happens in time as an effect of the long tail distribution.

### Results for the multi-disciplinary calibration

For the calibration of our second set of simulations, we use the data obtained from the second (peer review, multi-disciplinary) data extraction. In this case, we used a longer time scale, and a larger number of journals with respect to the previous one. Parameters are summed up in Table 3 (right). Note that, for the exponential fit, we recycle the value for the mono-dimensional case. This is because the fit, as shown in Fig. 4, is too bad to be considered.

**Fig. 7:**
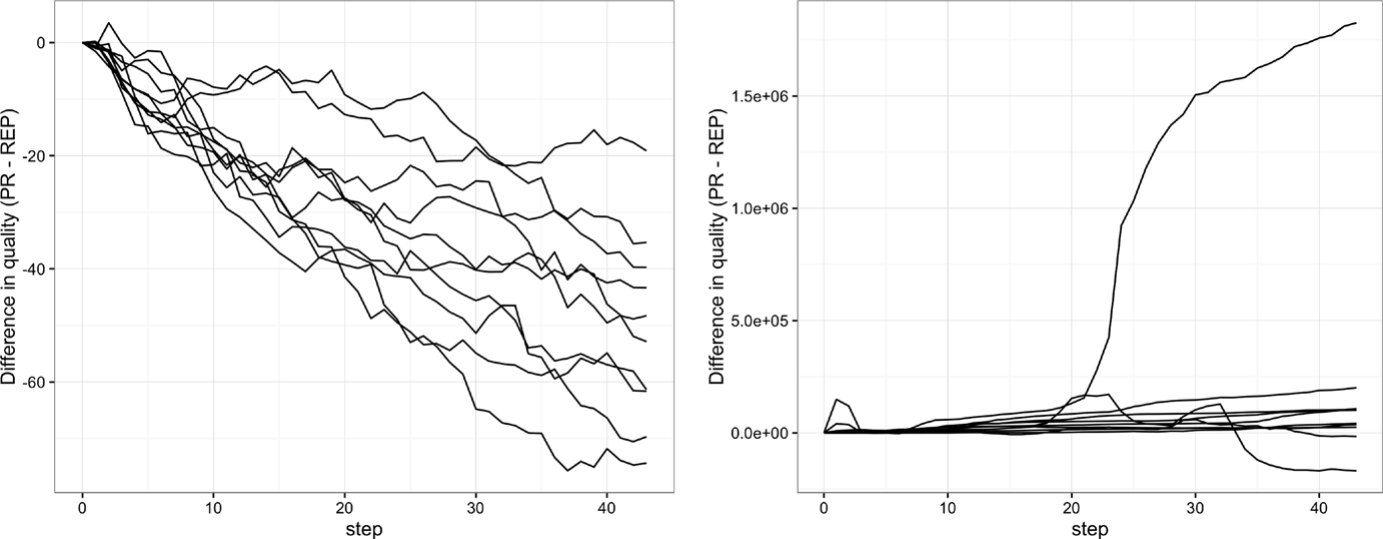
Results for the simulations calibrated using the peer review data extraction with different quality distributions: exponential (left) and Zipf (right)

We also run ten simulations and we show the difference in quality when applying the peer review and reputation sub-models to the same model of production as explained in Sect. 4.1. We present the evolution of this measure in the form of individual simulation stories, in Fig. 7, so that negative values mean that reputation reads result in more quality. This is again the case for the exponential scenario, where, apart from being slightly more jagged at visual analysis, reputation holds the upper hand in all cases. The Zipf scenario, instead, presents a surprise. The large events that pushed for reputation seem to go the opposite way, earning peer review a solid advantage on reputation.

**Fig. 8:**
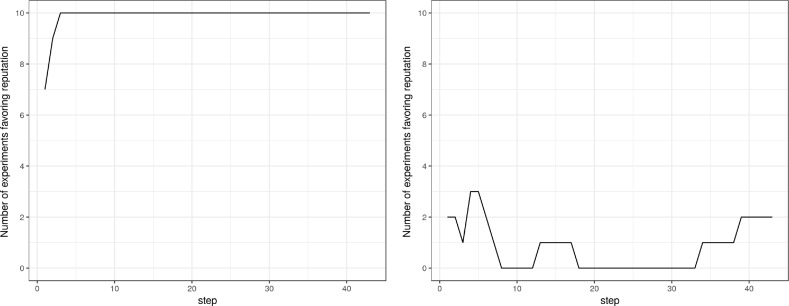
Number of simulations where reputation prevails in the multi-disciplinary case

This qualitative result is also evident in Fig. 8, where we show the number of simulations where the reputation mechanism is more efficient. This is true in both cases, although the exponential case gives a much clearer prevalence of reputation over peer review.

### Discussion

We have shown how our model can be calibrated to represent different publishing environments, such as a single journal (mono-disciplinary) or a wider set of publication outlets on the same subject (multi-disciplinary). The data extracted for calibration leave room for different assumptions on the underling quality structure, that, we remember, is accessed only through one of its manifestations: the number of citations.

In this work, we have chosen to use two alternative hypothesis on the underlying quality structure; the first, exponential, assumes a relatively flat quality landscape, with a strict upper limit to quality. The second, Zipf, allows for the emergence of large and unpredictable outliers—papers of exceptional quality, that mark the opening of a new field or revolutionize the field they appear in. The resulting landscape is highly irregular and comparing two histories, value by value, would be meaningless in this condition.

Our simulation model puts together and compares these two factors – the quality distribution and the disciplinary level—and to compare the effects of two different information filtering mechanisms: reputation and peer review.

The results are summed up in Table 4. The reputation mechanism prevails sharply, for both calibrations, in the case of exponential quality distribution. This suggests that peer review would not work as well as a standard reputation system in a flat landscape. The picture is different if we assume a Zipf quality distribution, that is, to an irregular, spiked landscape. Here, in the mono-disciplinary case, peer review prevails in the initial period, but reputation starts to perform better after 5 years. Subsequently, reputation prevails, although in a less clear way if we compare it to the exponential case. Note that the time trend would be relevant from the point of view of a decision maker: a decision made, for example, from the beginning of our time series and up to the time frame of an EU project (3 years), would be correct in the relatively flat exponential landscape, while it would be wrong in the present (mono-dimensional, Zipf) case.

**Table 4:** Values for the JAAMAS simulation

Quality distribution	Mono-disciplinary	Multi-disciplinary
Exponential	Reputation	Reputation
Zipf	Tie (Reputation prevails in the end)	Peer review

Lastly, we examine the multi-disciplinary scenario with a Zipf quality distribution. This combination gives rise to the complicated stories that can be seen in the right half of Fig. 7, with high spikes taking the difference between the two coupled runs to higher orders of magnitude. A pattern emerges only from the count of prevalence (Fig. 8, right), where the number of prevailing stories, at any moment in time, is largely favorable to peer review. While the result is clear from this perspective, if we look at the individual stories as an example of ten independent explorations, decision making would be much more difficult as one would easily end caught in local extremes.

A follow up question is, what was important and what was not in our parameter choice? In other words, how much generality we can assume in our results? We have checked variations of our main parameters, one at a time, in a $\pm\, 10\%$ range^5^ with no qualitative difference in the results. Further exploration, and a more in-depth study of parameter significance, are left for future work.

## Conclusions and outlook

In this paper we have achieved three goals. The first one was the construction of a model of science evaluation that could deal with different quality distributions. We have separated paper production from paper consumption, applying two different filters—peer review and reputation—so as to compare their effectiveness as information filters.

The second objective was to calibrate our model on real data. To this purpose, we performed two extractions from the Scopus database, one for a specific journal with a narrow disciplinary scope (JAAMAS) and the other addressing a potentially large and diverse community, as nearly all the fields of science have something to say about peer review. Calibration helped us setting the research question in a realistic context. Instead of running simulations across a parameter space too large to explore, calibration allowed us to situate our main research question—what is the role of outliers with respect to different information filter mechanisms—namely, reputation and peer review. This was our third objective.

Our results show that in the case of a specific discipline, reputation is better than traditional peer review to optimize the quality of papers read by researchers. A single journal does not perform well in an information filtering role. Of course, acting as a filter is not the only purpose of a journal; it could be playing several different roles, as helping the community to stick together, and increasing the quality of papers through a re-submission process (Cowley 2015), all elements not yet included in our model.

In the case of a wider disciplinary subject as peer review itself, the scenario proposed from our simulation changes. If the field is either mature or incremental, that is, if the quality landscape is relatively flat, also in this case (as in the previous one) a reputation system could be sufficient to maximize quality. Instead, if we allow outliers within the wider disciplinary subject, peer review is more effective. Stretching the metaphor, we could say that our simulation would support the transition to a reputation system for normal science, but would suggest sticking to peer review for multi-disciplinary exceptional science.

## Electronic supplementary material

Below is the link to the electronic supplementary material.

Supplementary material 1 (zip 5147 KB)

## Footnotes

1 The model is availabe on GitHub at https://github.com/LABSS/PeerReviewGPS.

2 to preserve dimensionality, this noise is calculates as $e_i*e_j/q_r$ where $e_{i,j}$ are two extracted by the distribution selected for quality, and $q_r$ is the quality of the reviewer.

3 https://www.scopus.com.

4 Published by Springer, edited by Carles Sierra and Michael Luck. ISSN: 1387-2532.

5 Specifically, in Online Resouce 1 we have checked $\pm 10\%$ percentage variations on the productivity and Zipf exponent parameters, and unit ($\pm\,1$) variations on the number of journals.
