# Supplementary material for: Reputation or peer review? The role of outliers
Source: Scientometrics. 2018 Jul 9;116(3):1421–38. doi: 10.1007/s11192-018-2826-3 (PMC6096687; doi:10.1007/s11192-018-2826-3)
Supplement: Supplementary file 1 — Supplementary material 1 (zip 5147 KB) [file 11192_2018_2826_MOESM1_ESM.zip › ESM_1/ESM_1.pdf]

1 Robustness check of the reported results

The figures below prove the robustness of the reported results when varing three key parameters of the model: scientist productivity (Figures 1 and 2), quality distribution (Figures 3 and 4) and number of journals (Figures 5 and 6). The plots show no qualitative difference with the results presented in the paper.

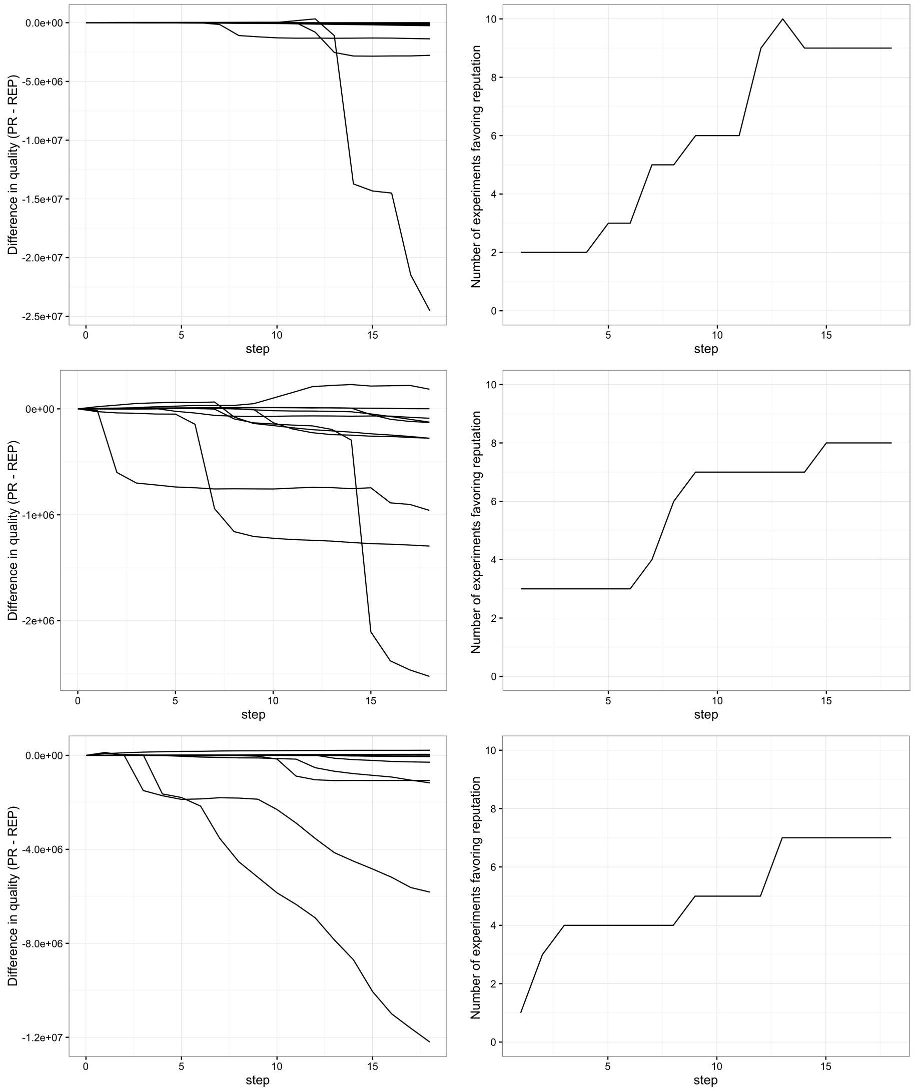

Figure 1: Variation on mean productivity (mono-disciplinary case, 10 runs): 0.15 (middle row, baseline), 0.135 (top row, −10%), 0.165 (bottom row, +10%)

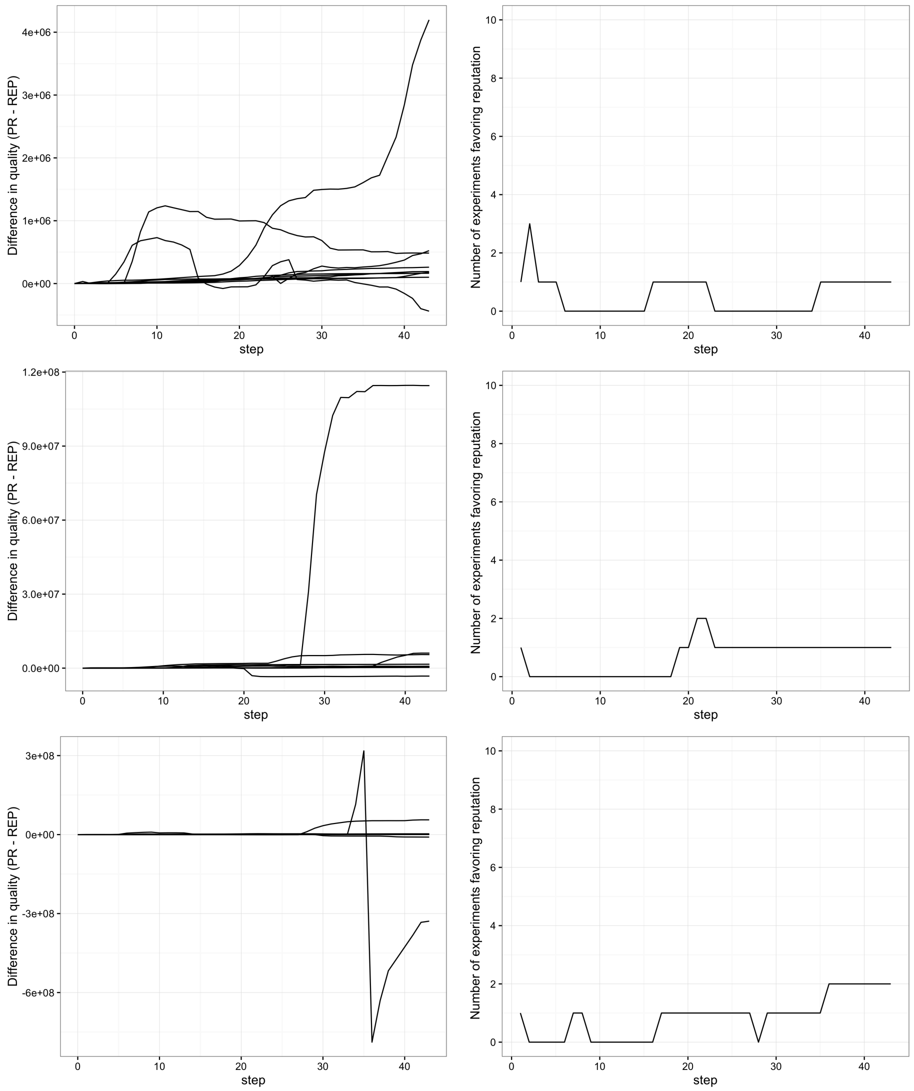

Figure 2: Variation on mean productivity (multi-disciplinary case, 10 runs): 0.07 (middle row, baseline), 0.063 (top row, −10%), 0.077 (bottom row, +10%)

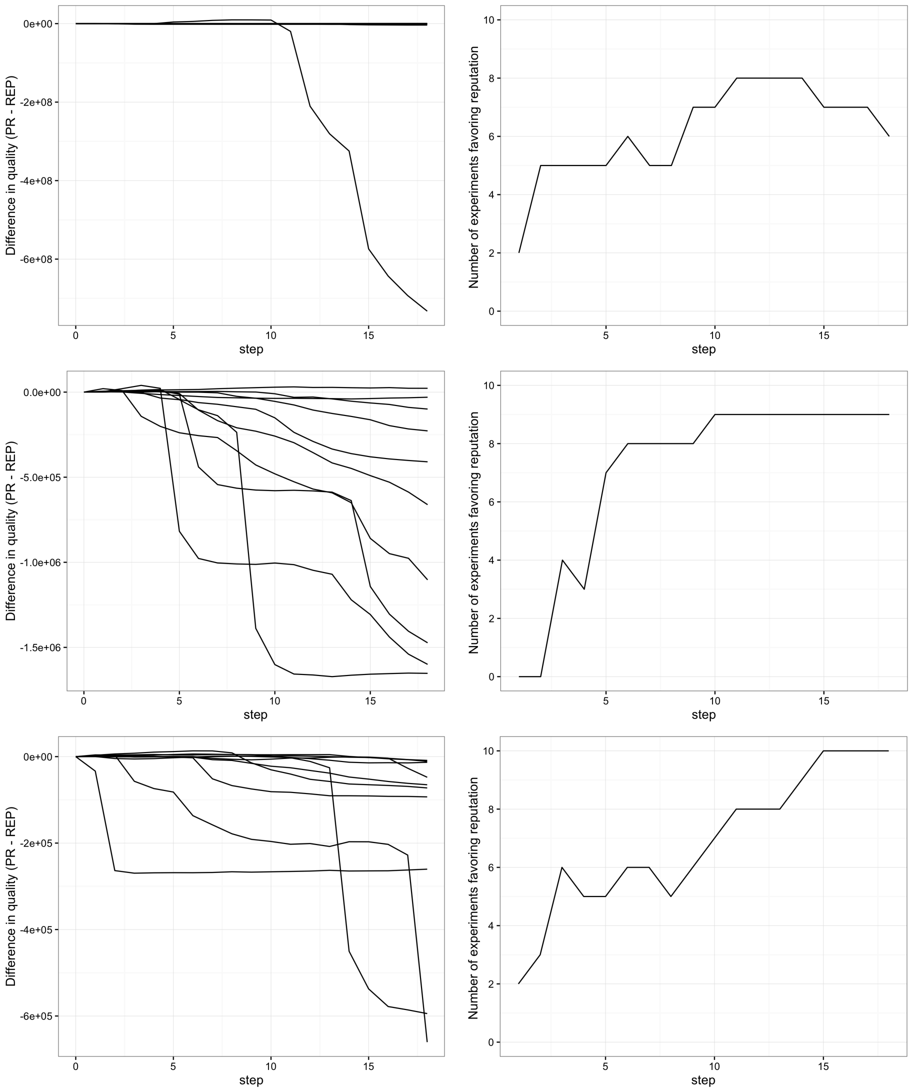

Figure 3: Variation on the Zipf exponent (mono-disciplinary case, 10 runs): 1.87 (middle row, baseline), 1.683 (top row, −10%), 2.057 (bottom row, +10%)

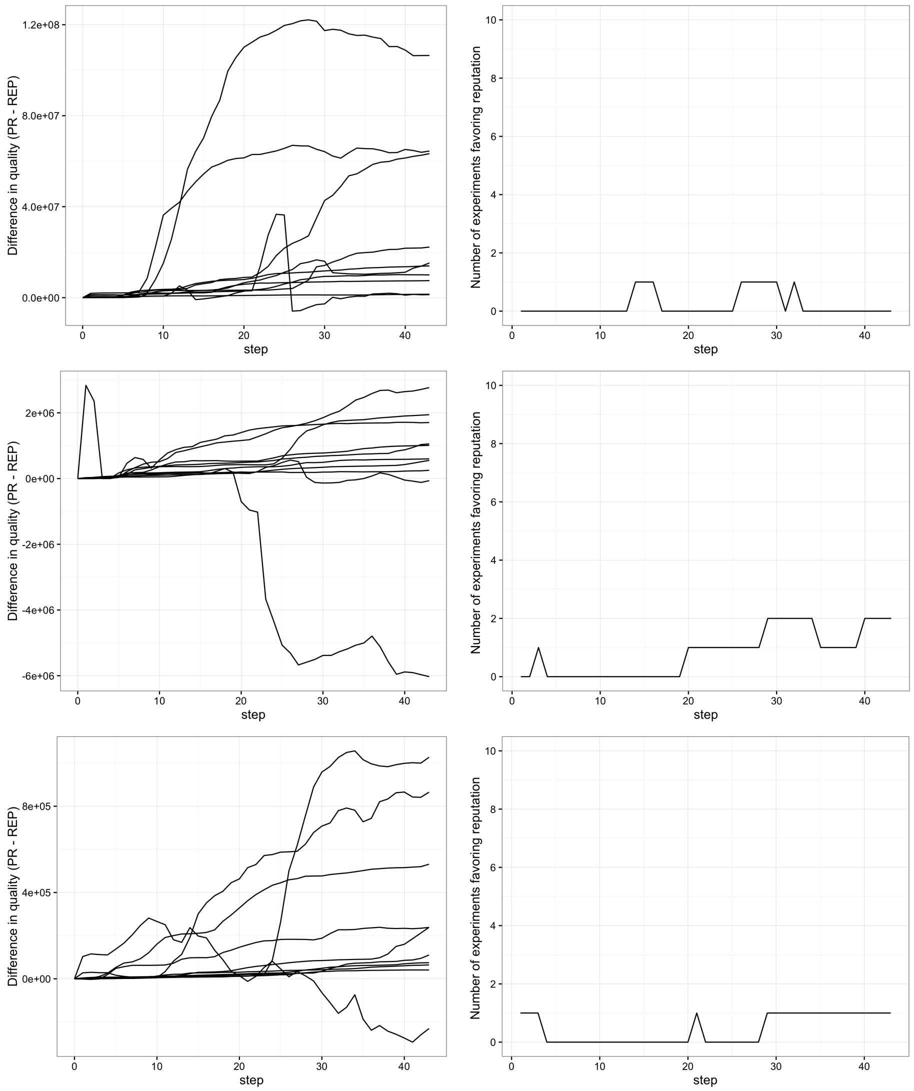

Figure 4: Variation on the Zipf exponent (multi-disciplinary case, 10 runs): 1.72 (middle row, baseline), 1.548 (top row, −10%), 1.892 (bottom row, +10%)

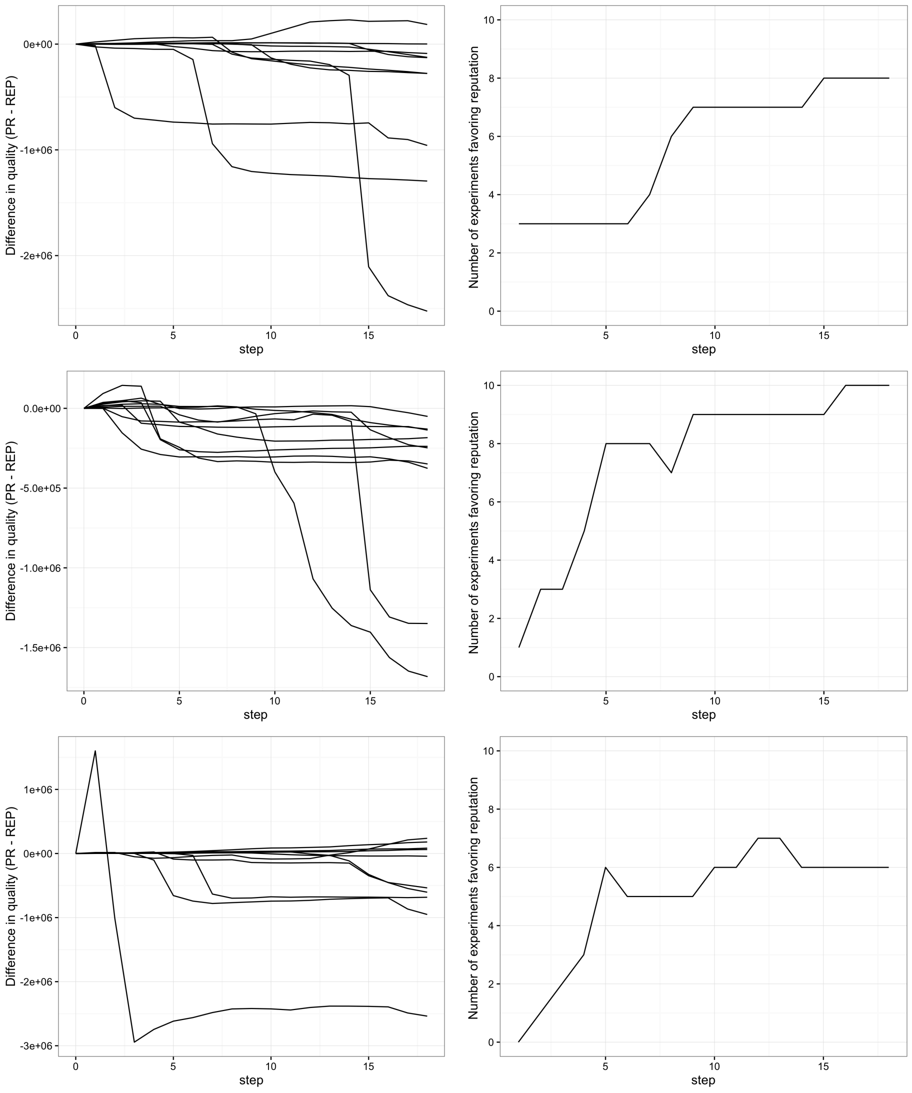

Figure 5: Variation on the number of journals (mono-disciplinary case, 10 runs): 1 journal (top row, baseline), 2 journals (middle row), 4 journals (bottom row)

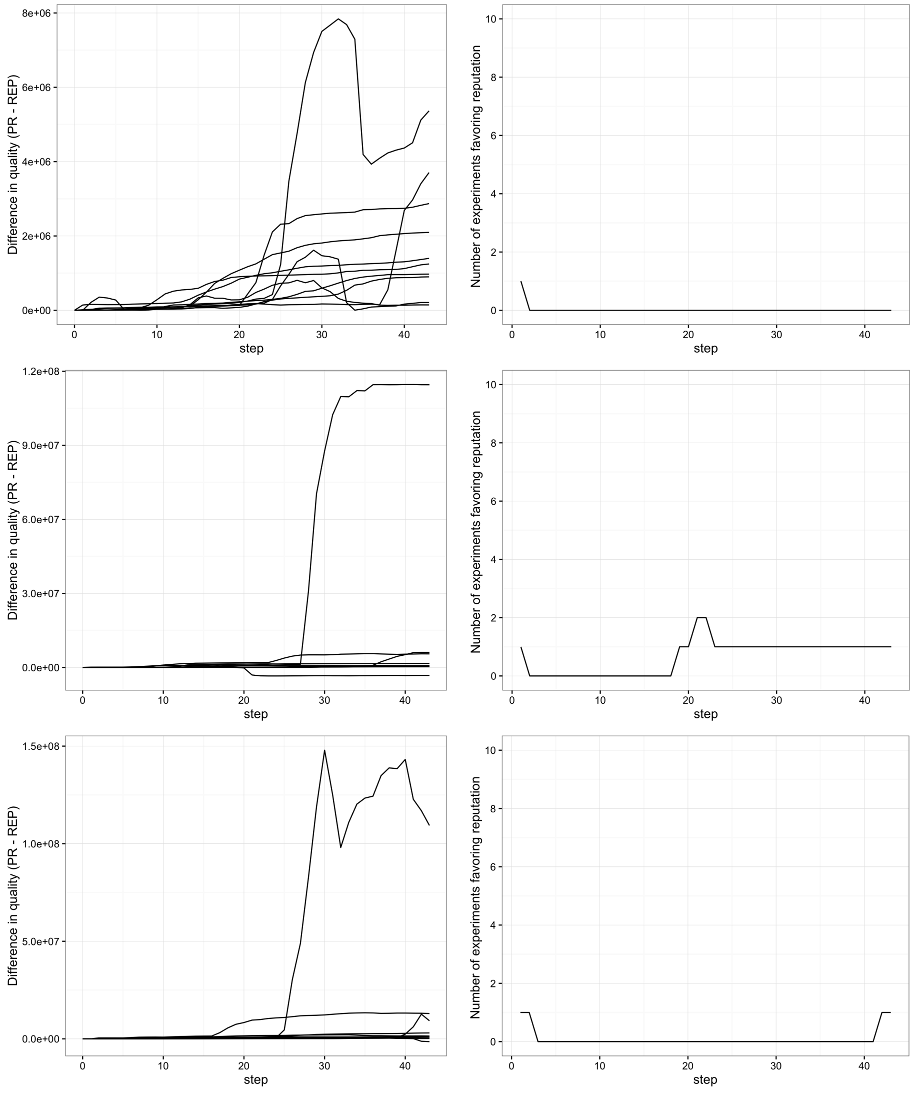

Figure 6: Variation on the number of journals (multi-disciplinary case, 10 runs): 36 journals (middle row, baseline), 35 journals (top row), 36 journals (bottom row)
