# Supplementary figures and images for: Reputation or peer review? The role of outliers
Source: Scientometrics. 2018 Jul 9;116(3):1421–38. doi: 10.1007/s11192-018-2826-3 (PMC6096687; doi:10.1007/s11192-018-2826-3)

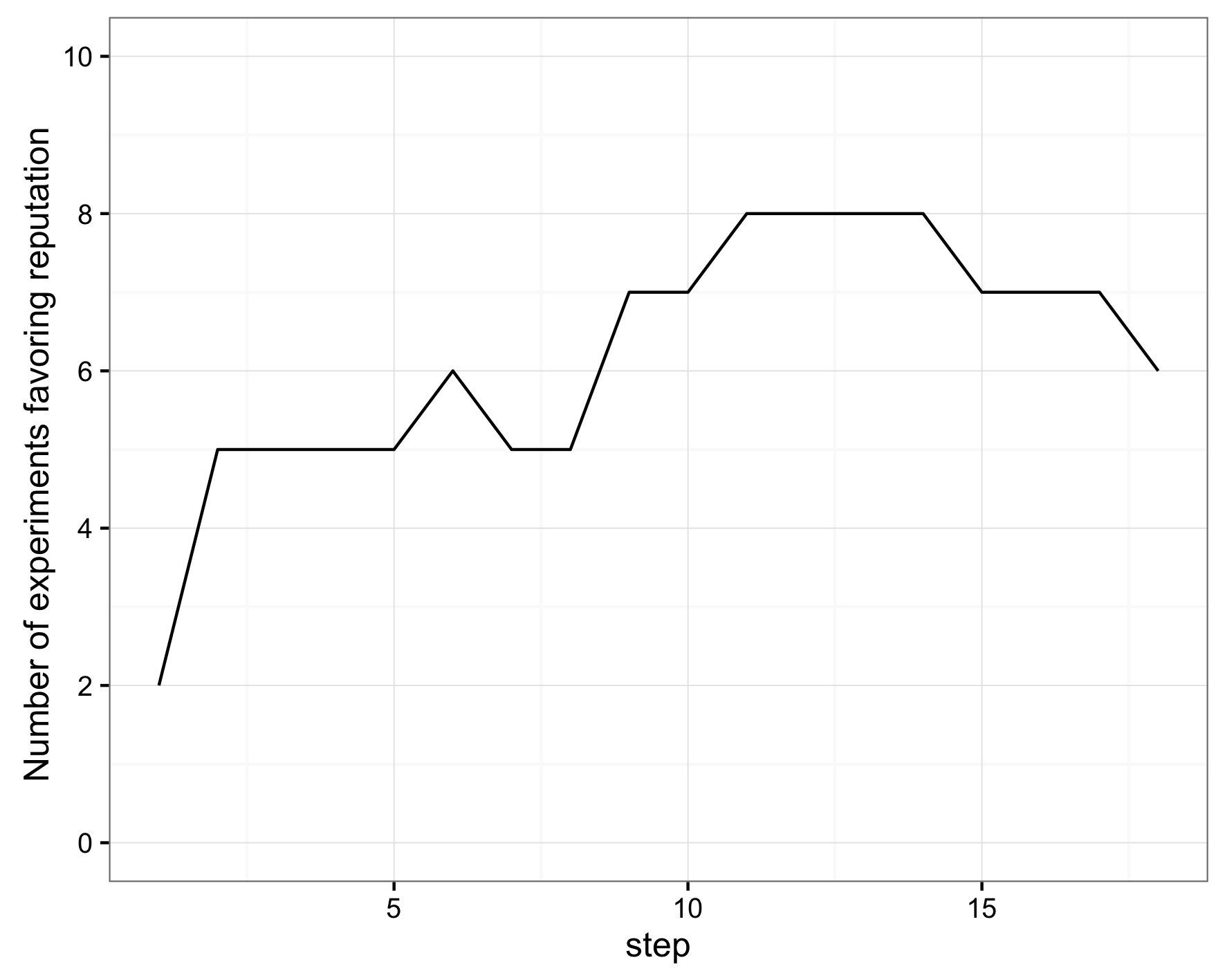

Supplement: Supplementary file 1 — Supplementary material 1 (zip 5147 KB) [file 11192_2018_2826_MOESM1_ESM.zip › ESM_1/v7-JAAMAS-zipf-41_z1683_diff_sim.png]

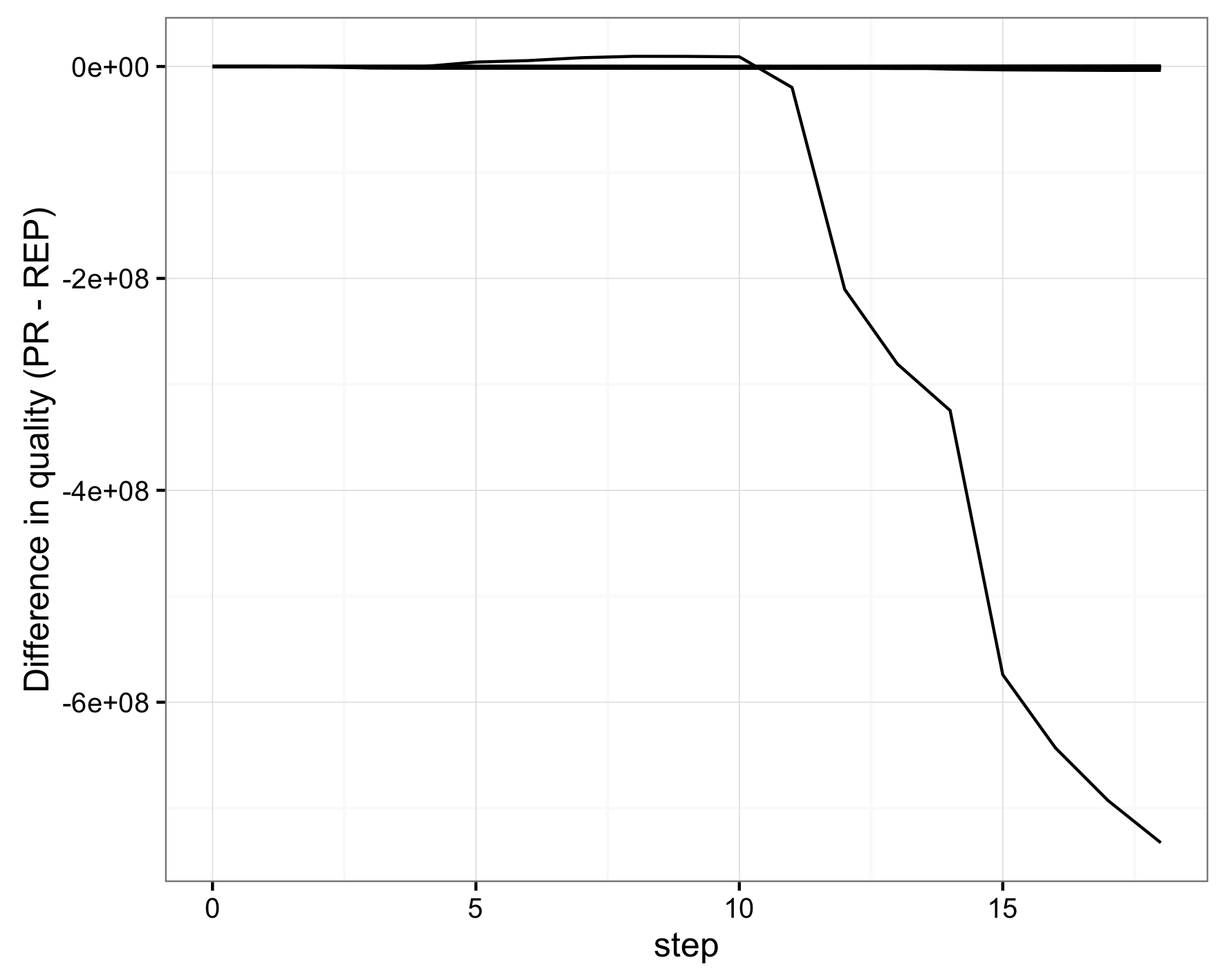

Supplement: Supplementary file 1 — Supplementary material 1 (zip 5147 KB) [file 11192_2018_2826_MOESM1_ESM.zip › ESM_1/v7-JAAMAS-zipf-41_z1683_sim.png]

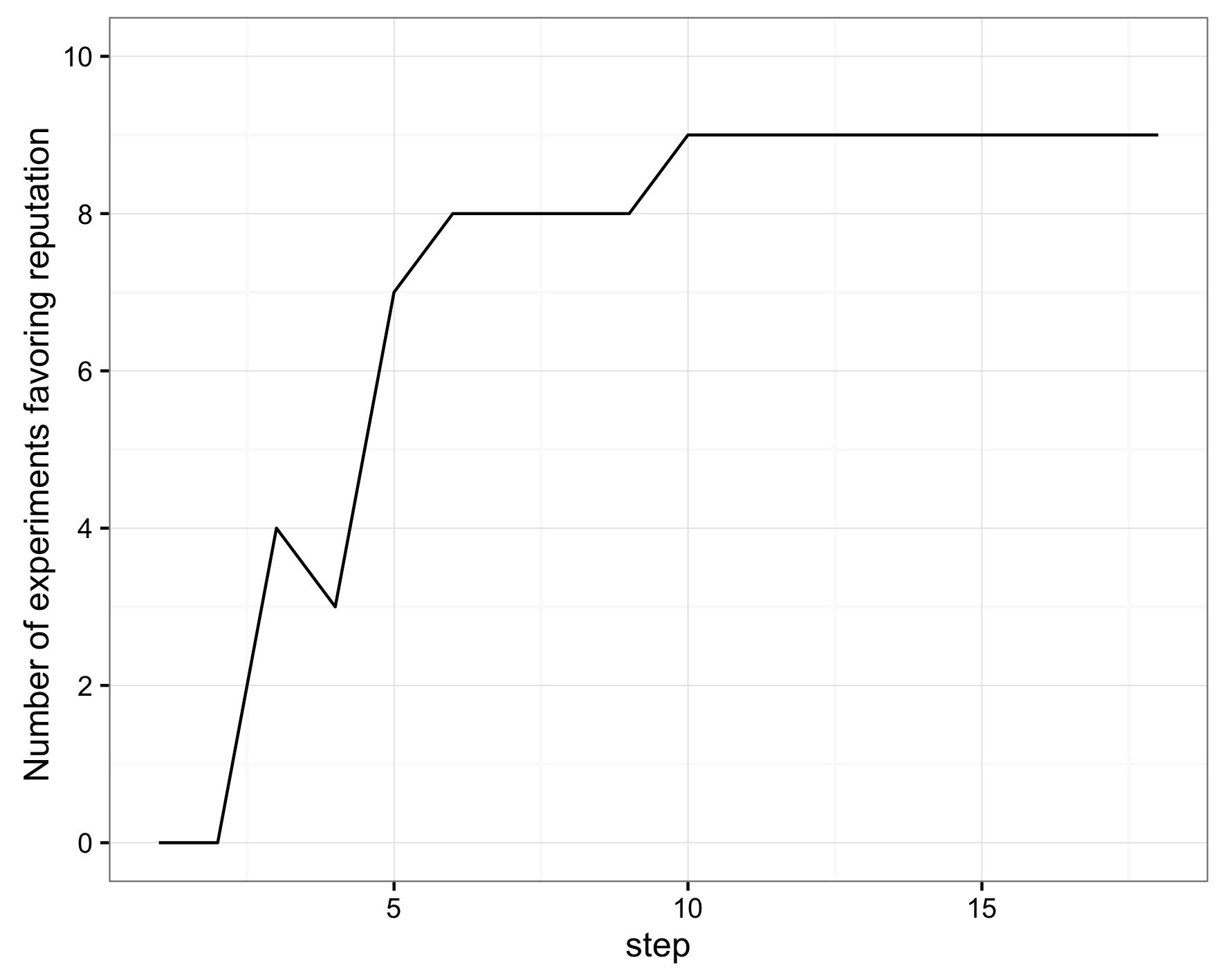

Supplement: Supplementary file 1 — Supplementary material 1 (zip 5147 KB) [file 11192_2018_2826_MOESM1_ESM.zip › ESM_1/v7-JAAMAS-zipf-41_z187_diff_sim.png]

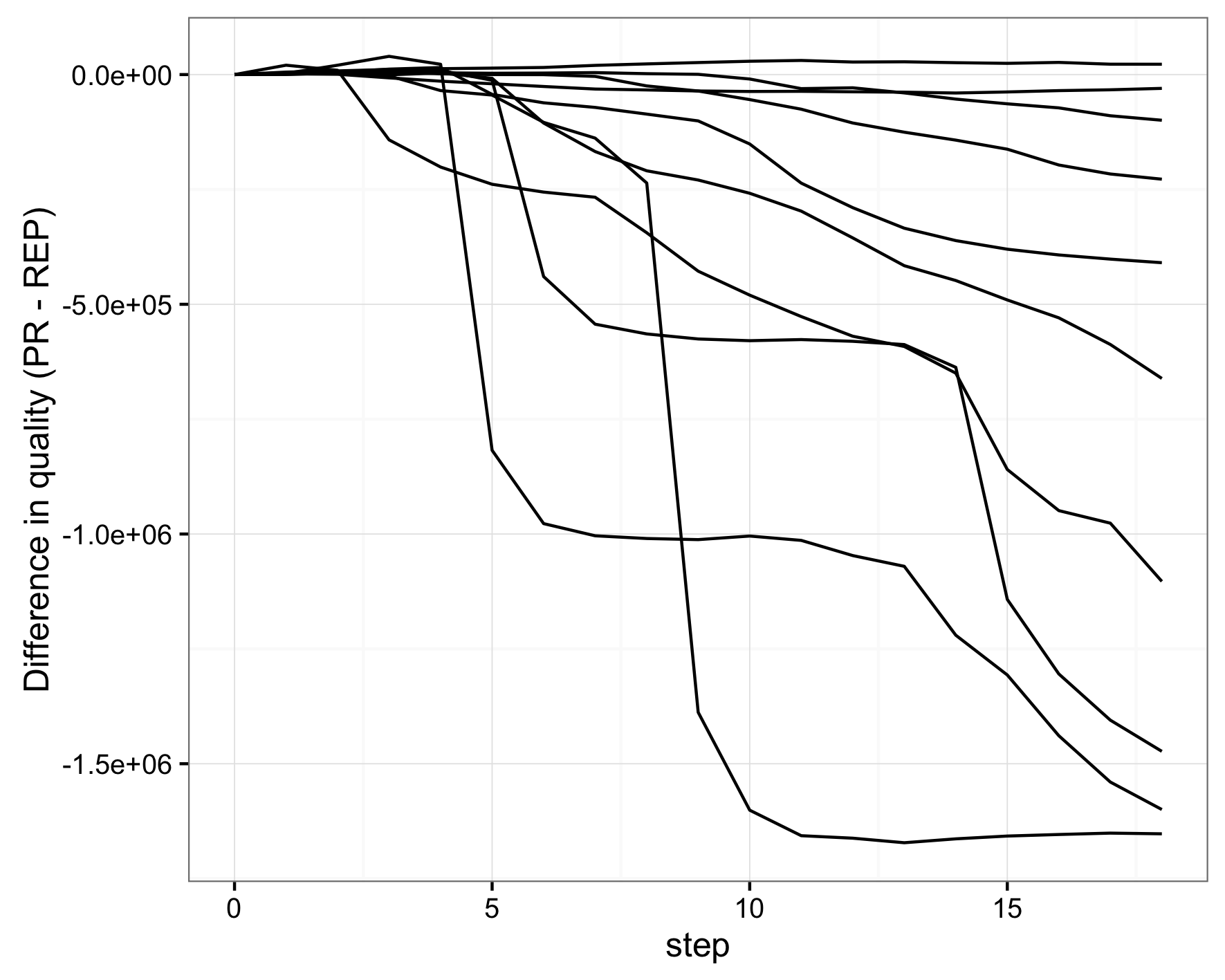

Supplement: Supplementary file 1 — Supplementary material 1 (zip 5147 KB) [file 11192_2018_2826_MOESM1_ESM.zip › ESM_1/v7-JAAMAS-zipf-41_z187_sim.png]

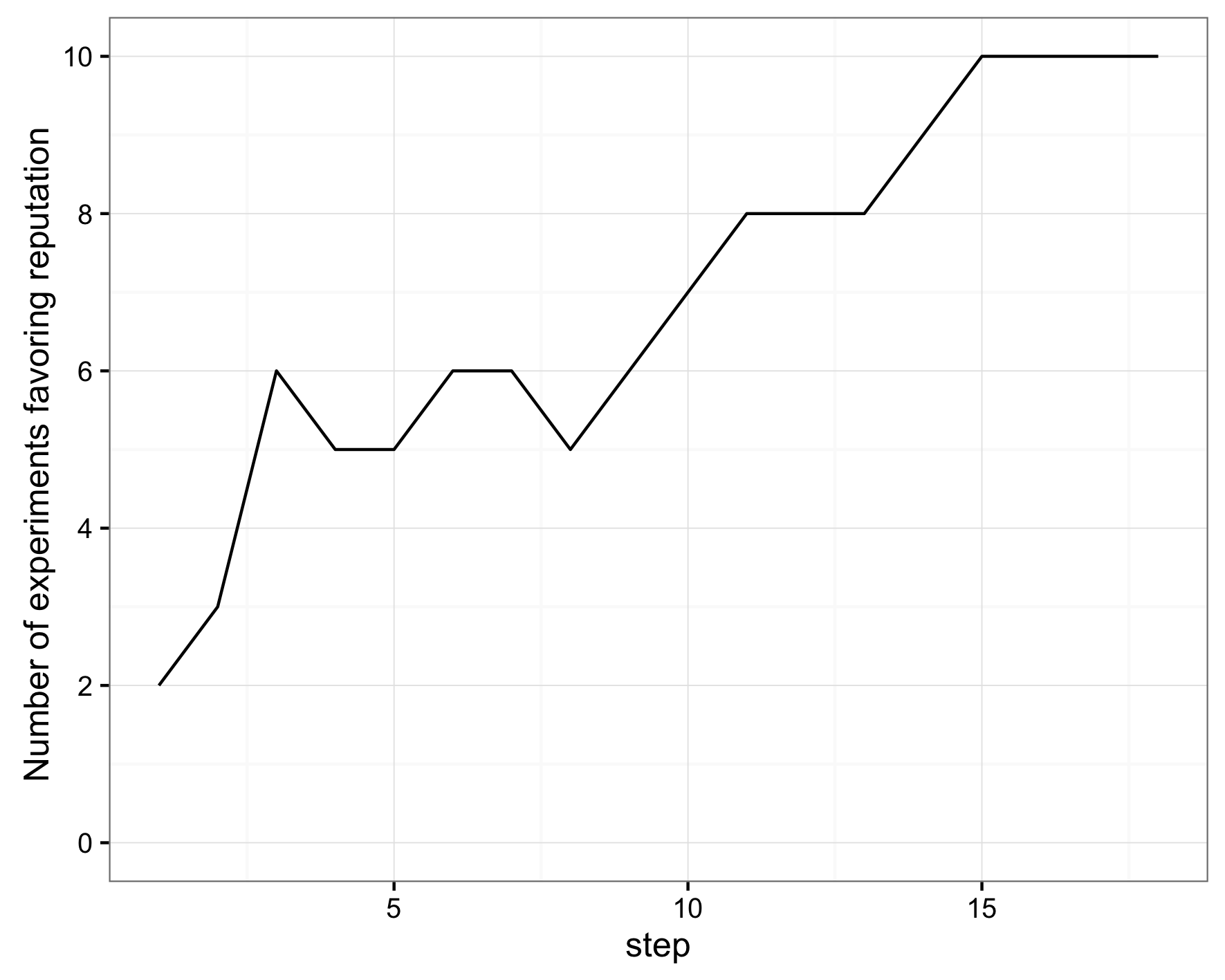

Supplement: Supplementary file 1 — Supplementary material 1 (zip 5147 KB) [file 11192_2018_2826_MOESM1_ESM.zip › ESM_1/v7-JAAMAS-zipf-41_z2057_diff_sim.png]

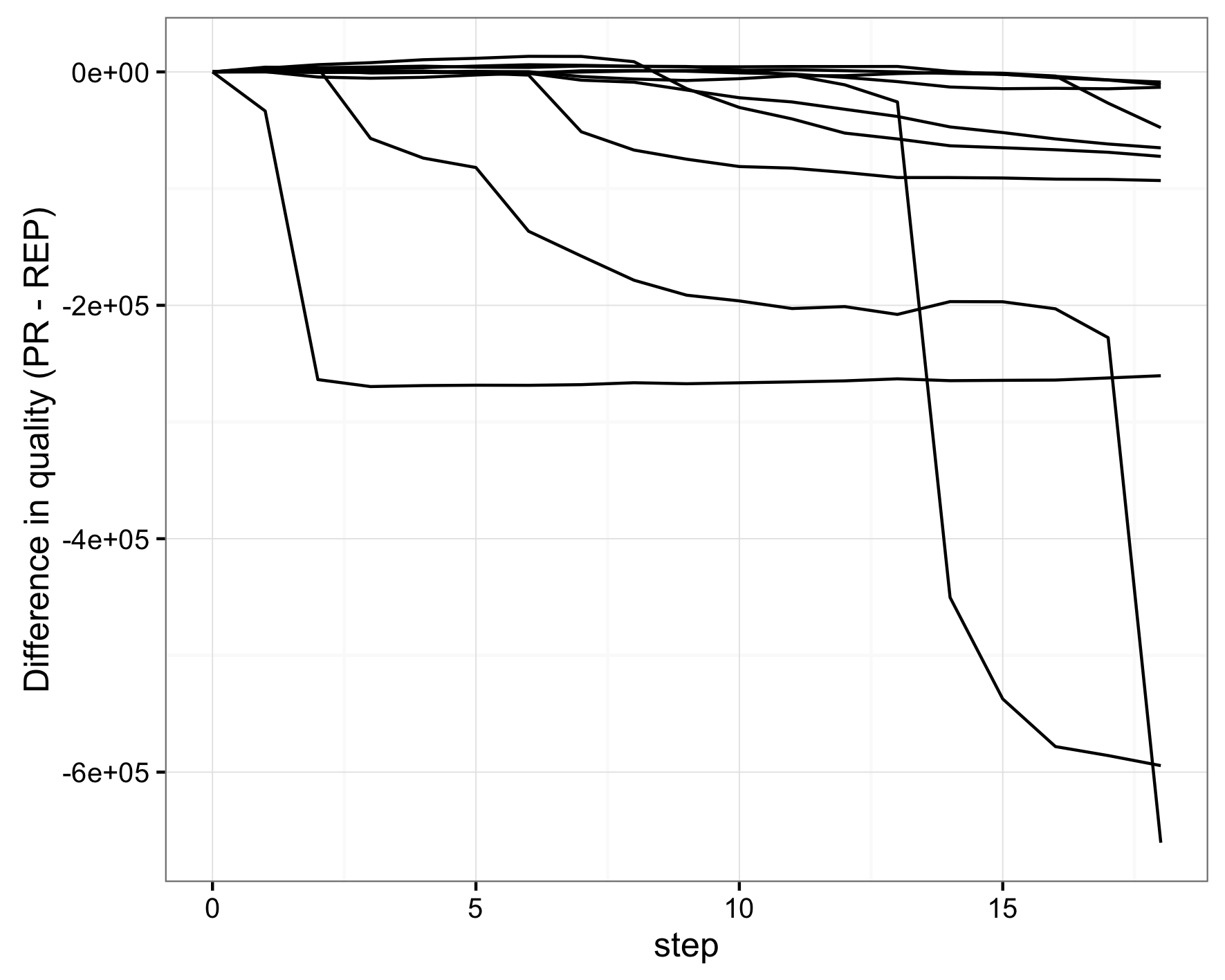

Supplement: Supplementary file 1 — Supplementary material 1 (zip 5147 KB) [file 11192_2018_2826_MOESM1_ESM.zip › ESM_1/v7-JAAMAS-zipf-41_z2057_sim.png]

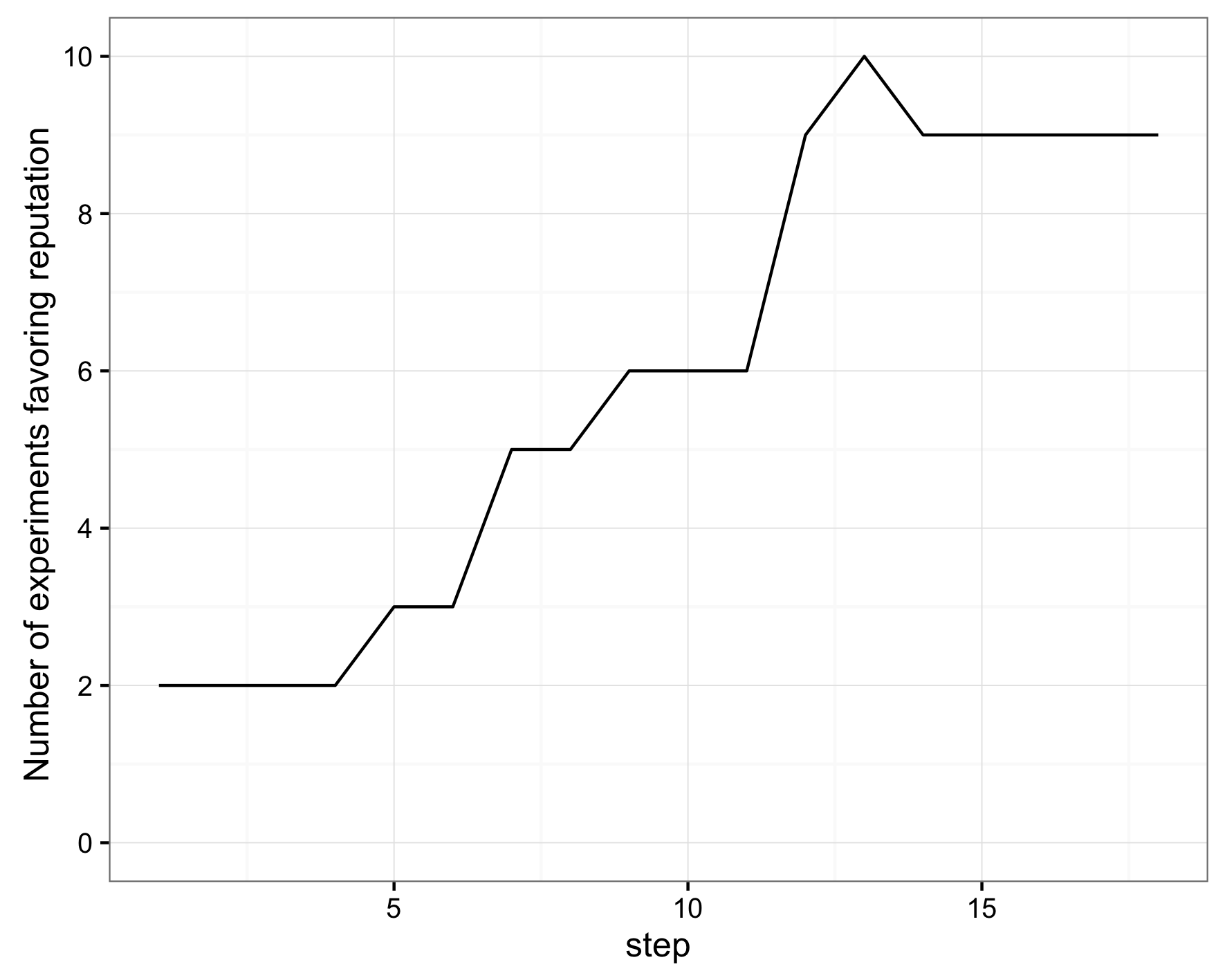

Supplement: Supplementary file 1 — Supplementary material 1 (zip 5147 KB) [file 11192_2018_2826_MOESM1_ESM.zip › ESM_1/v7-JAAMAS-zipf-43_p135_diff_sim.png]

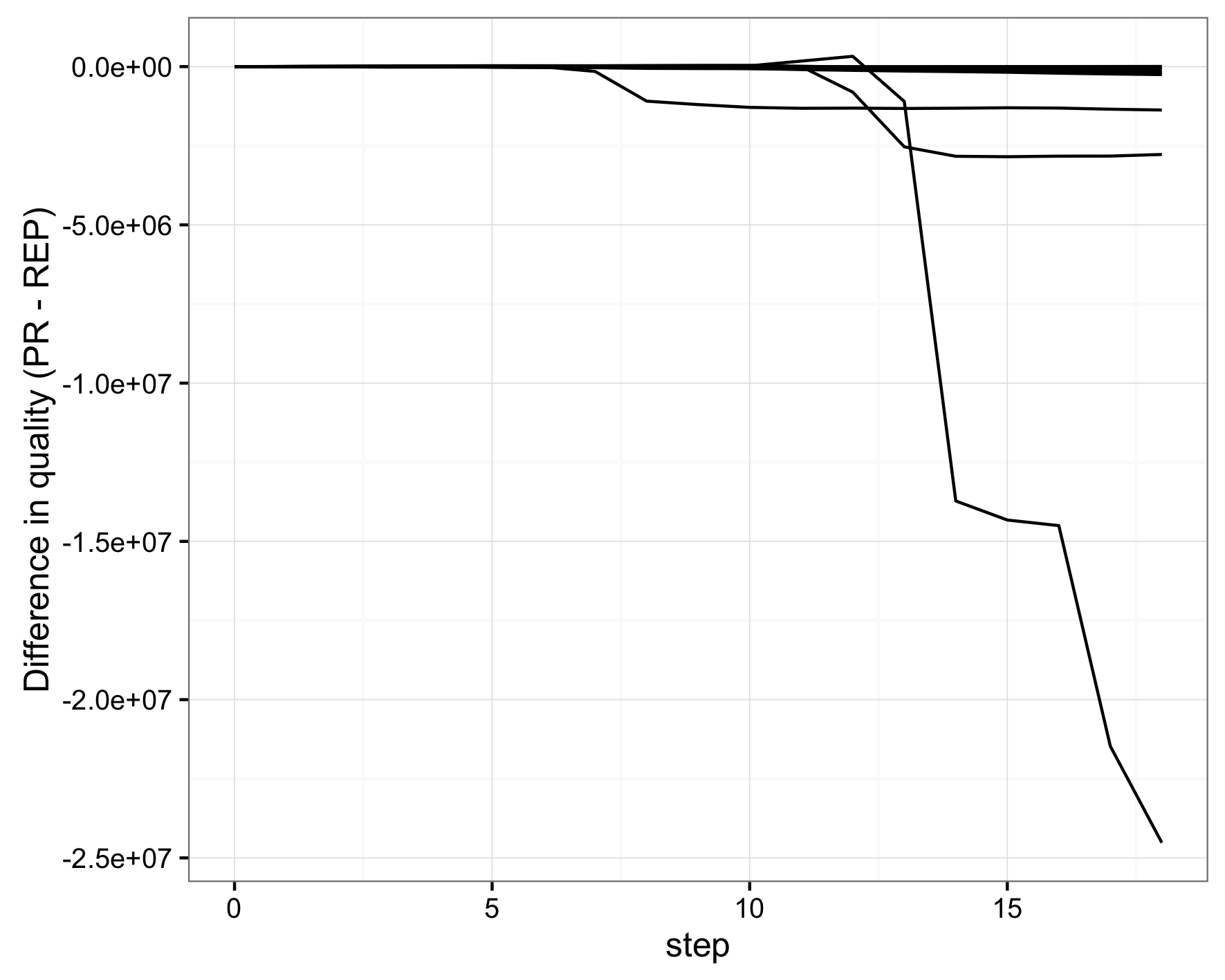

Supplement: Supplementary file 1 — Supplementary material 1 (zip 5147 KB) [file 11192_2018_2826_MOESM1_ESM.zip › ESM_1/v7-JAAMAS-zipf-43_p135_sim.png]

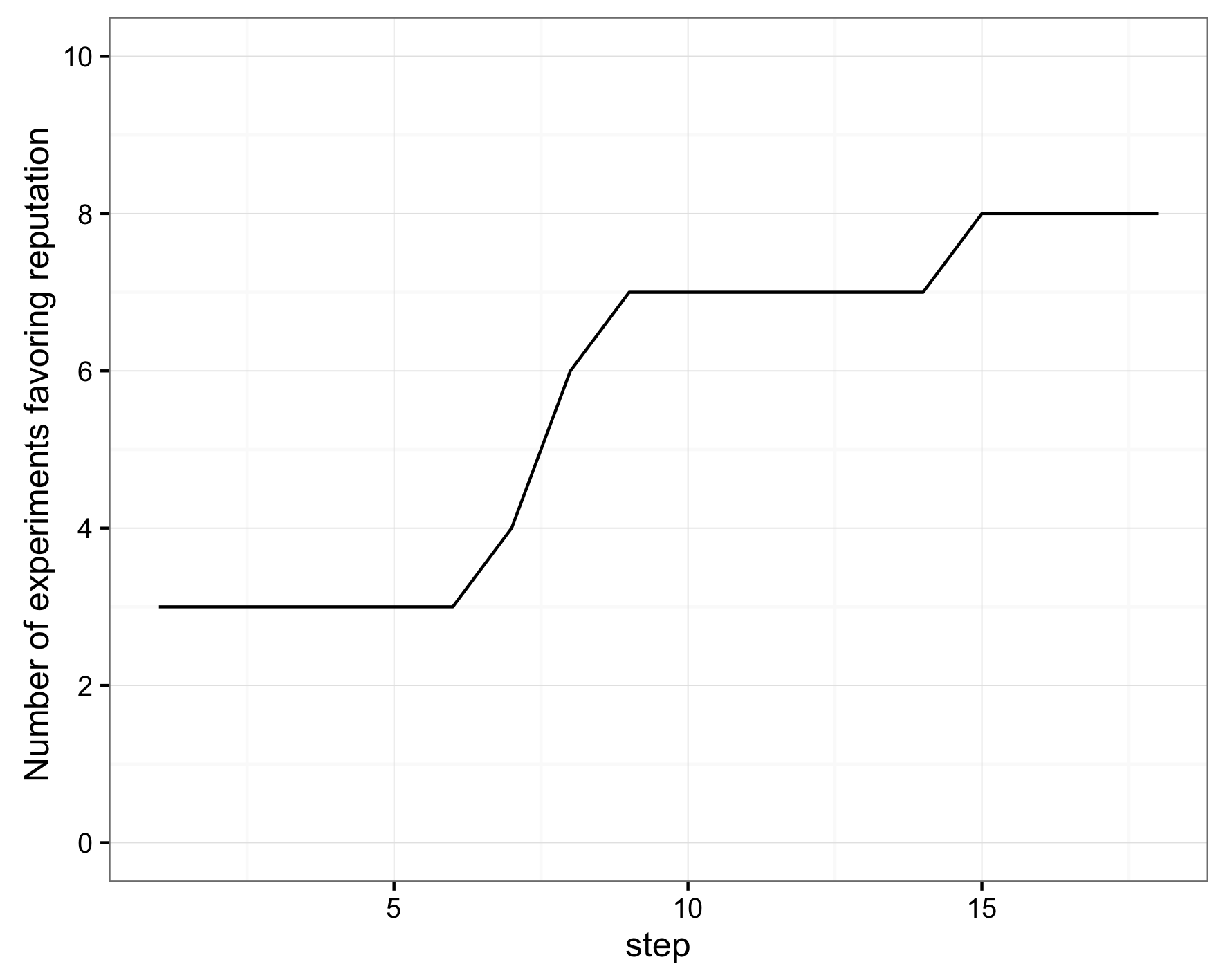

Supplement: Supplementary file 1 — Supplementary material 1 (zip 5147 KB) [file 11192_2018_2826_MOESM1_ESM.zip › ESM_1/v7-JAAMAS-zipf-43_p15_diff_sim.png]

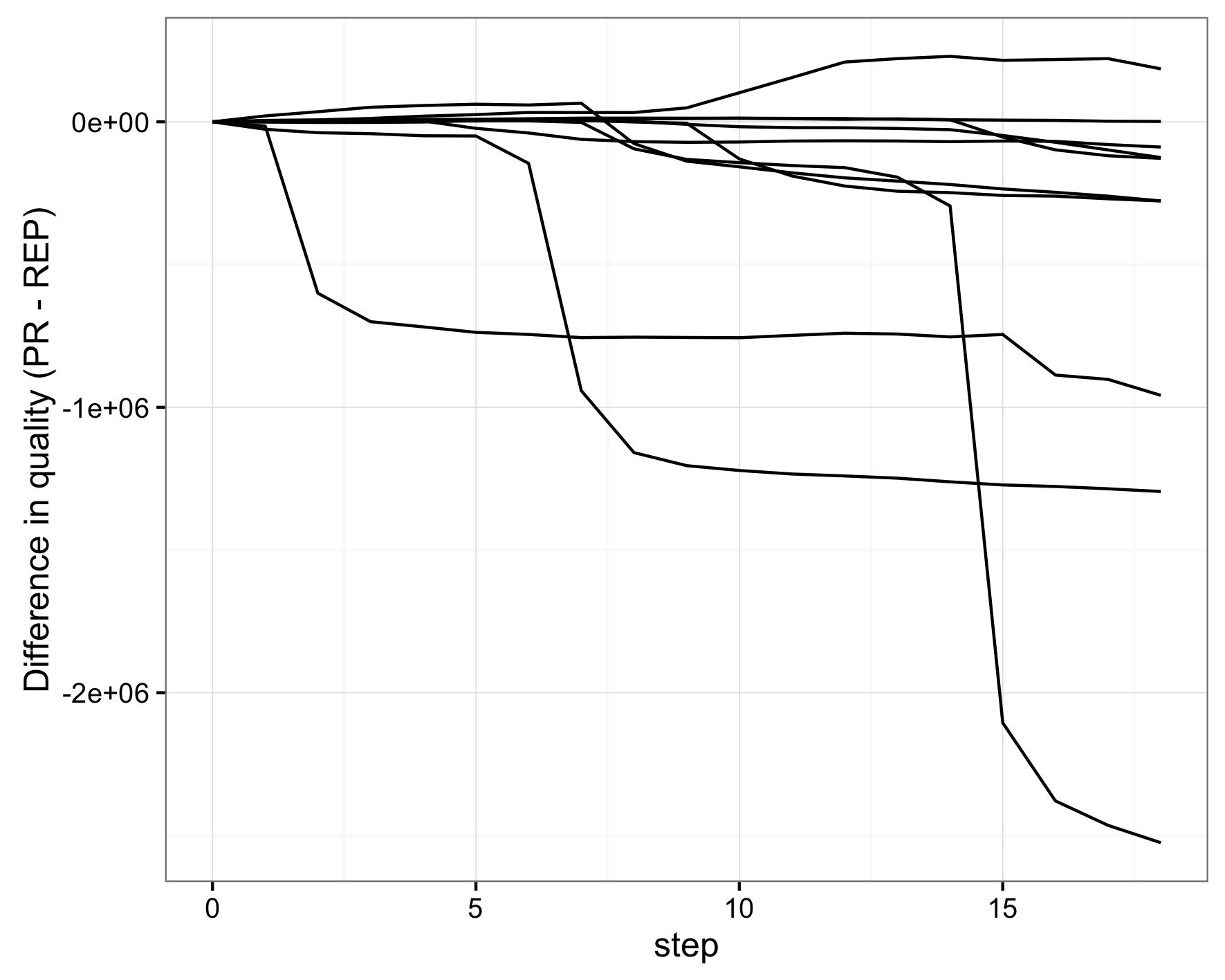

Supplement: Supplementary file 1 — Supplementary material 1 (zip 5147 KB) [file 11192_2018_2826_MOESM1_ESM.zip › ESM_1/v7-JAAMAS-zipf-43_p15_sim.png]

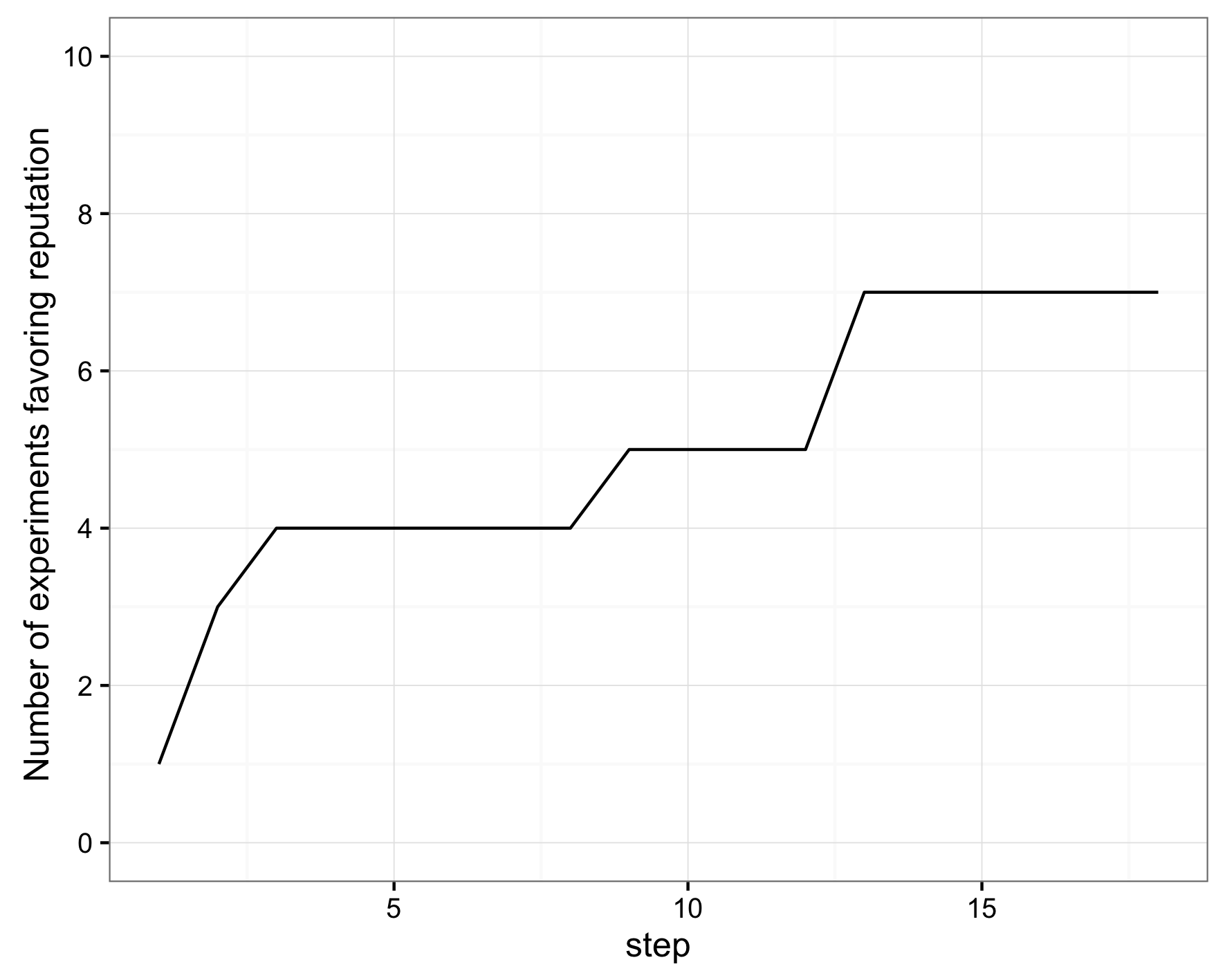

Supplement: Supplementary file 1 — Supplementary material 1 (zip 5147 KB) [file 11192_2018_2826_MOESM1_ESM.zip › ESM_1/v7-JAAMAS-zipf-43_p165_diff_sim.png]

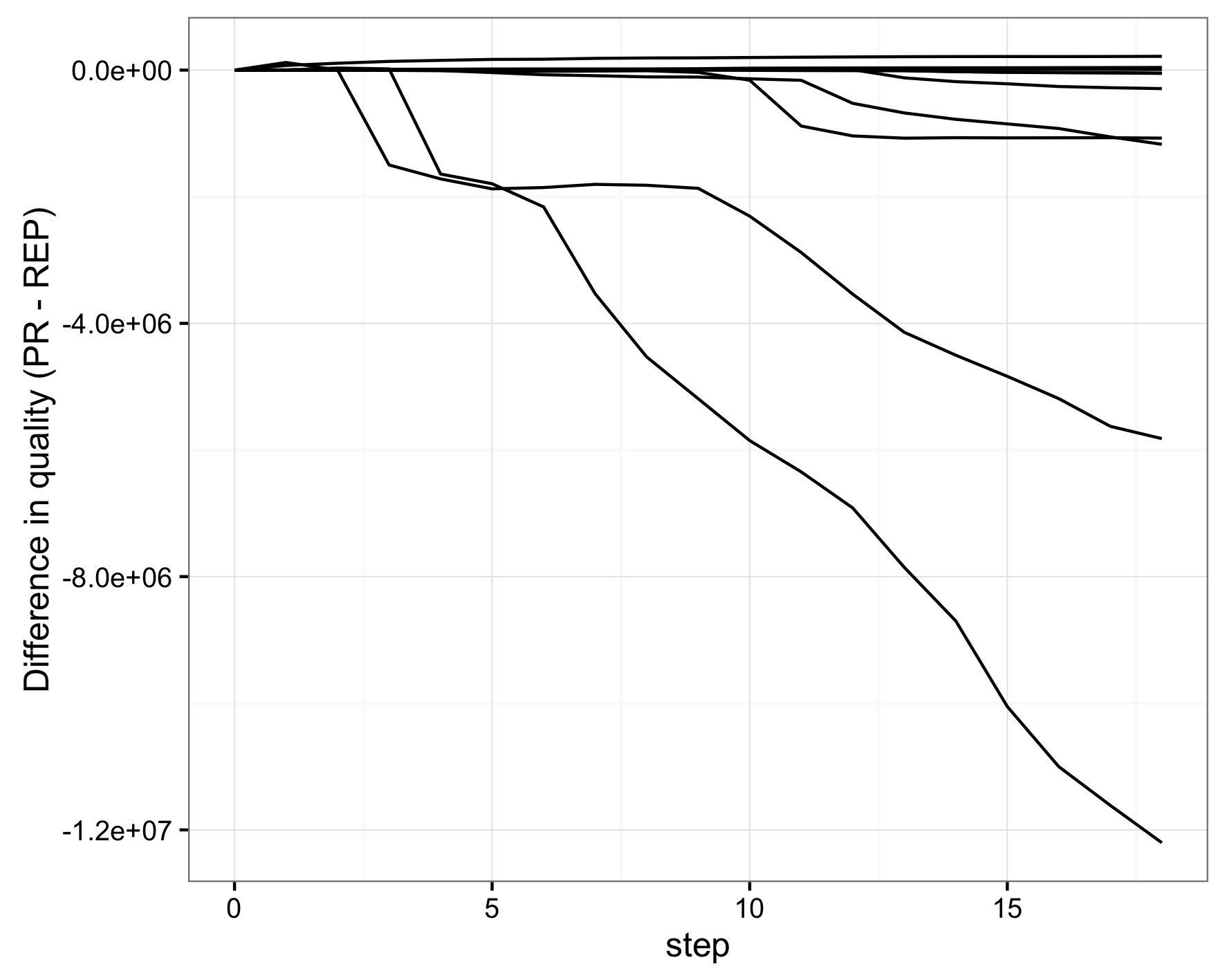

Supplement: Supplementary file 1 — Supplementary material 1 (zip 5147 KB) [file 11192_2018_2826_MOESM1_ESM.zip › ESM_1/v7-JAAMAS-zipf-43_p165_sim.png]

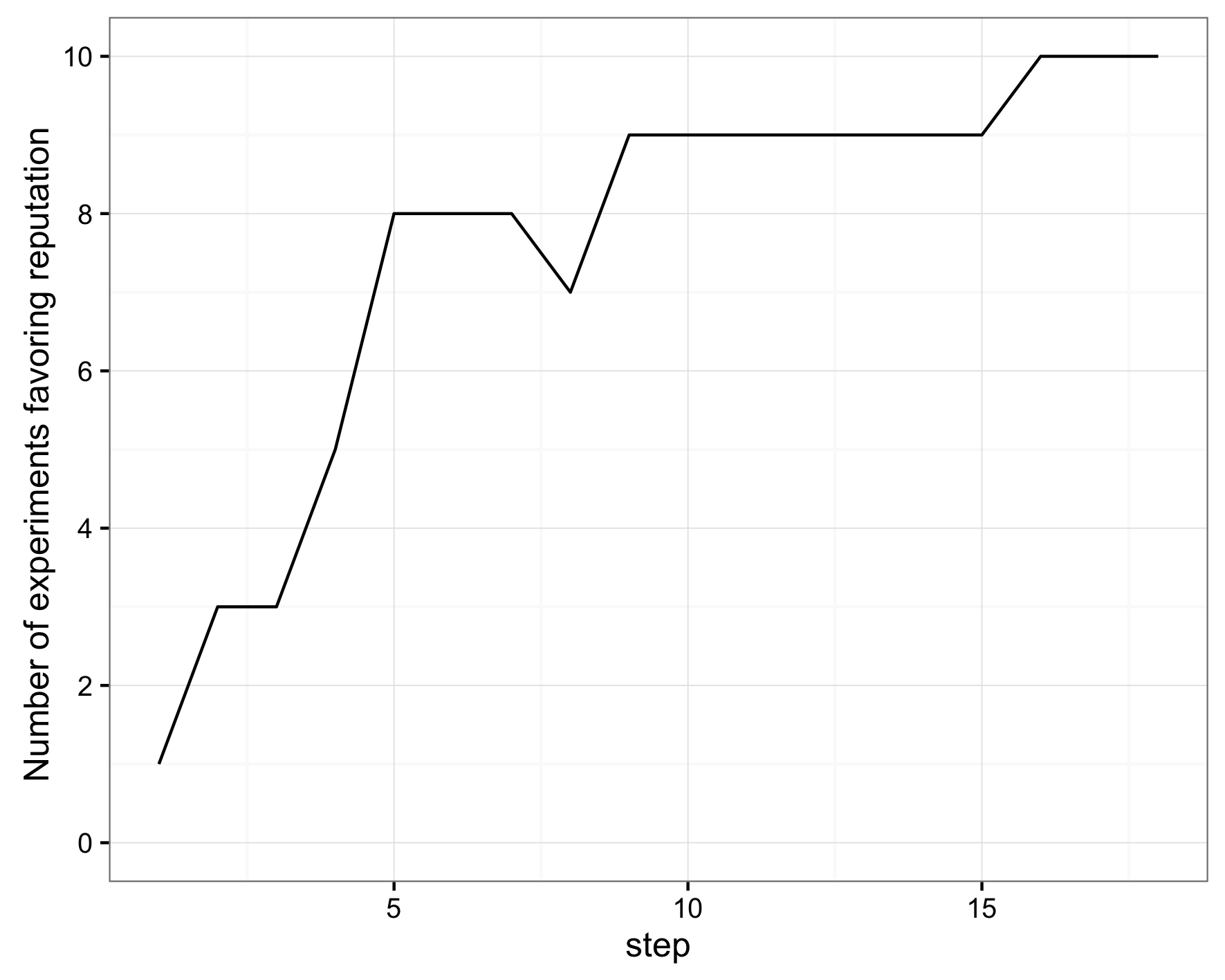

Supplement: Supplementary file 1 — Supplementary material 1 (zip 5147 KB) [file 11192_2018_2826_MOESM1_ESM.zip › ESM_1/v7-JAAMAS-zipf-45_j2_diff_sim.png]

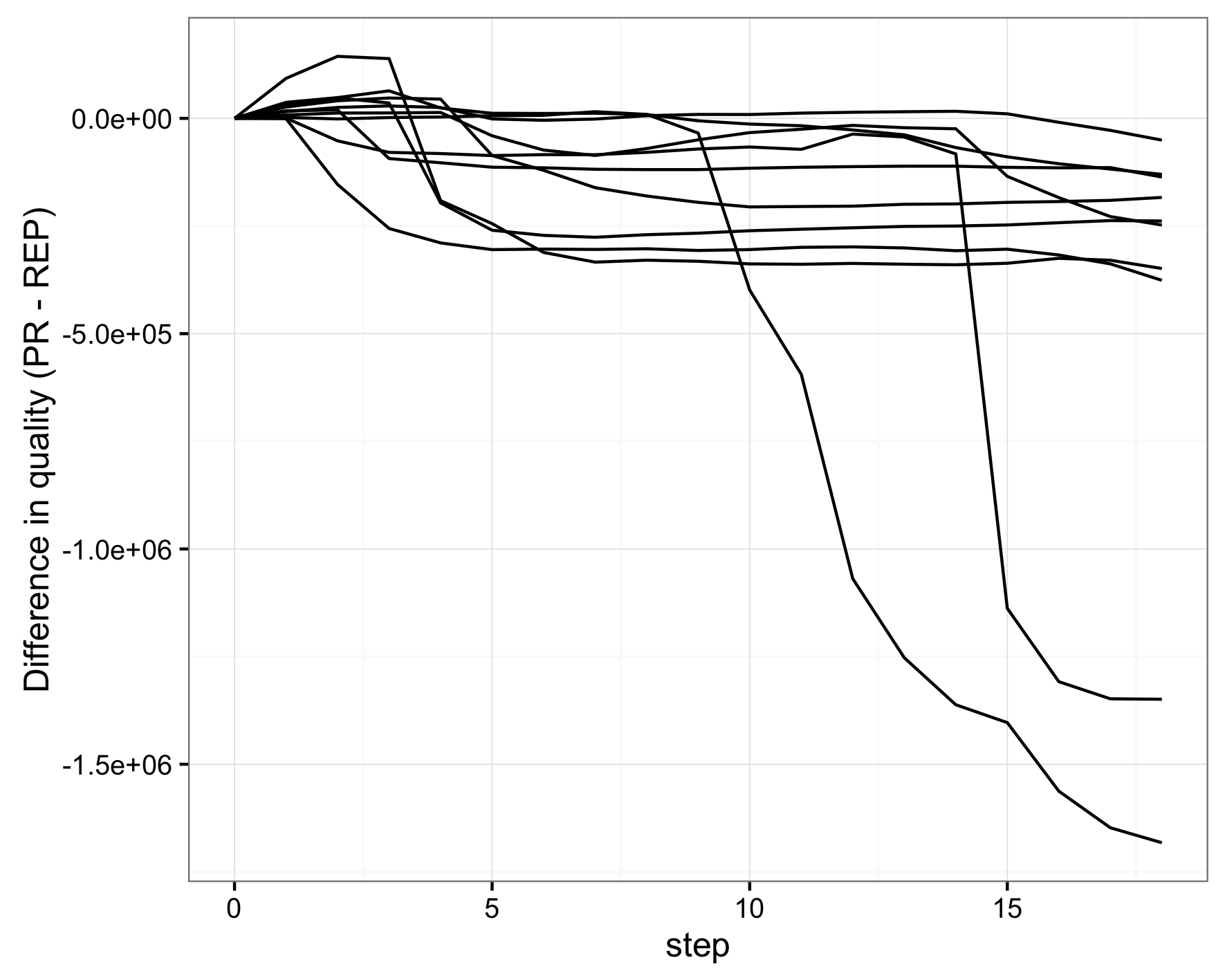

Supplement: Supplementary file 1 — Supplementary material 1 (zip 5147 KB) [file 11192_2018_2826_MOESM1_ESM.zip › ESM_1/v7-JAAMAS-zipf-45_j2_sim.png]

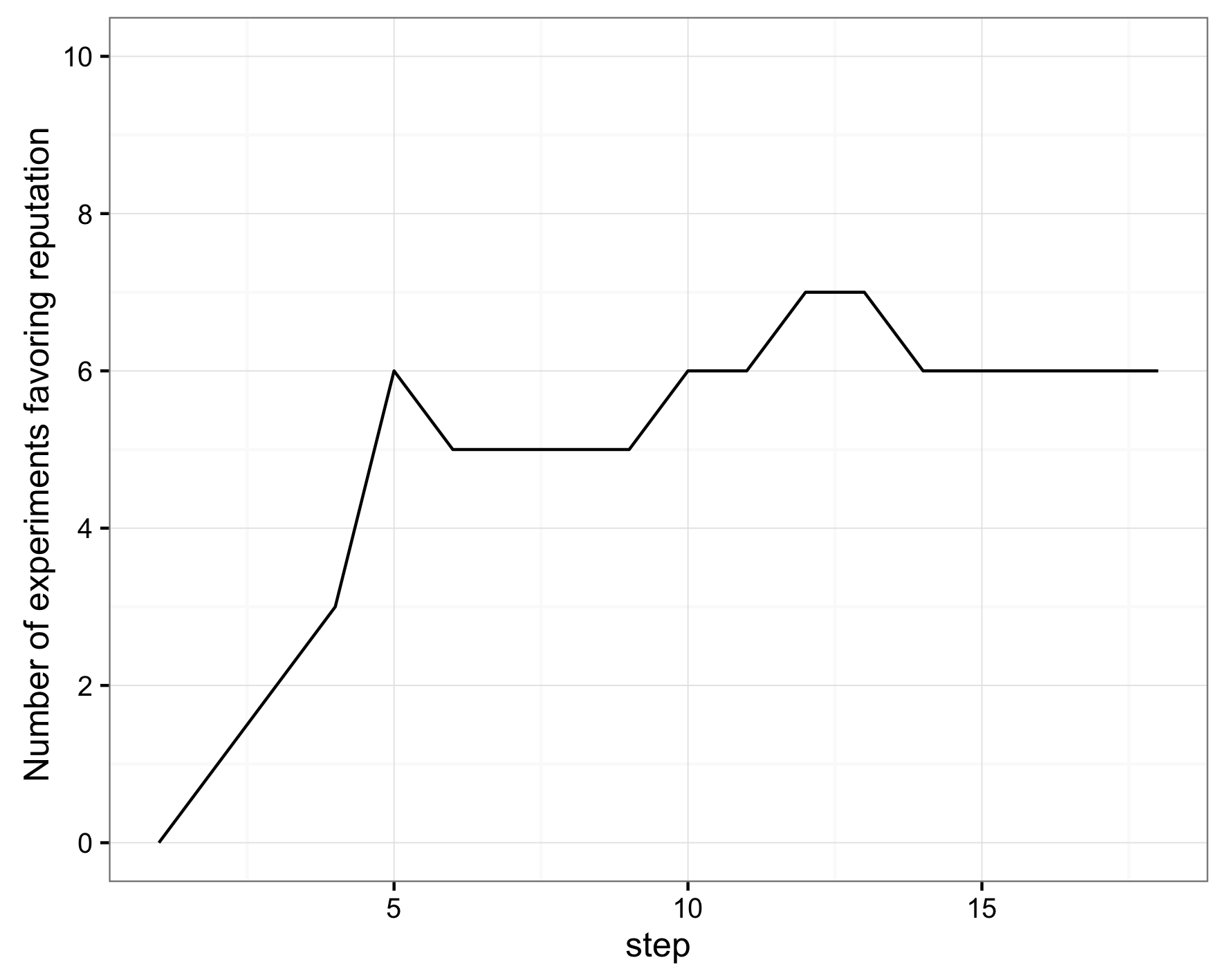

Supplement: Supplementary file 1 — Supplementary material 1 (zip 5147 KB) [file 11192_2018_2826_MOESM1_ESM.zip › ESM_1/v7-JAAMAS-zipf-45_j4_diff_sim.png]

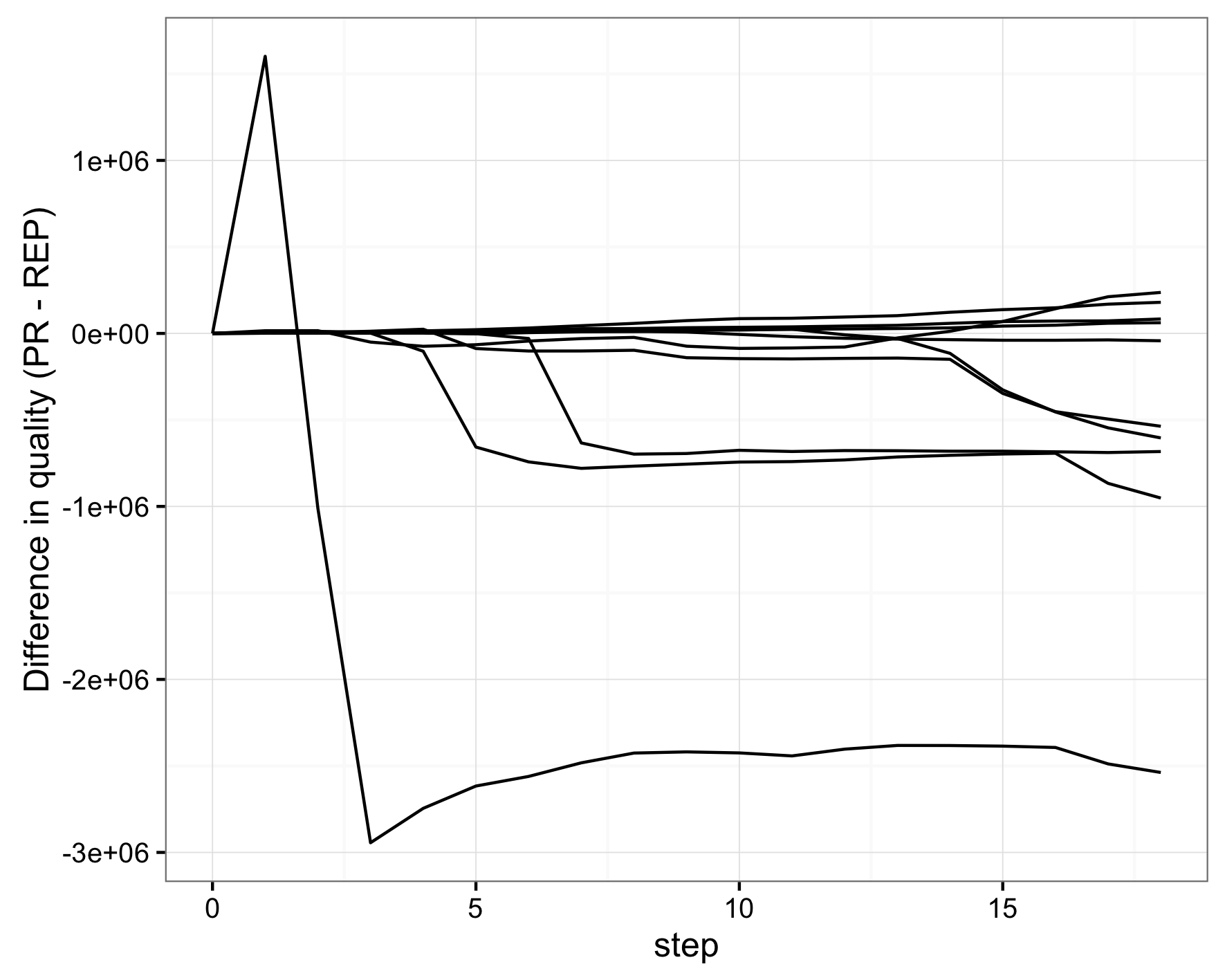

Supplement: Supplementary file 1 — Supplementary material 1 (zip 5147 KB) [file 11192_2018_2826_MOESM1_ESM.zip › ESM_1/v7-JAAMAS-zipf-45_j4_sim.png]

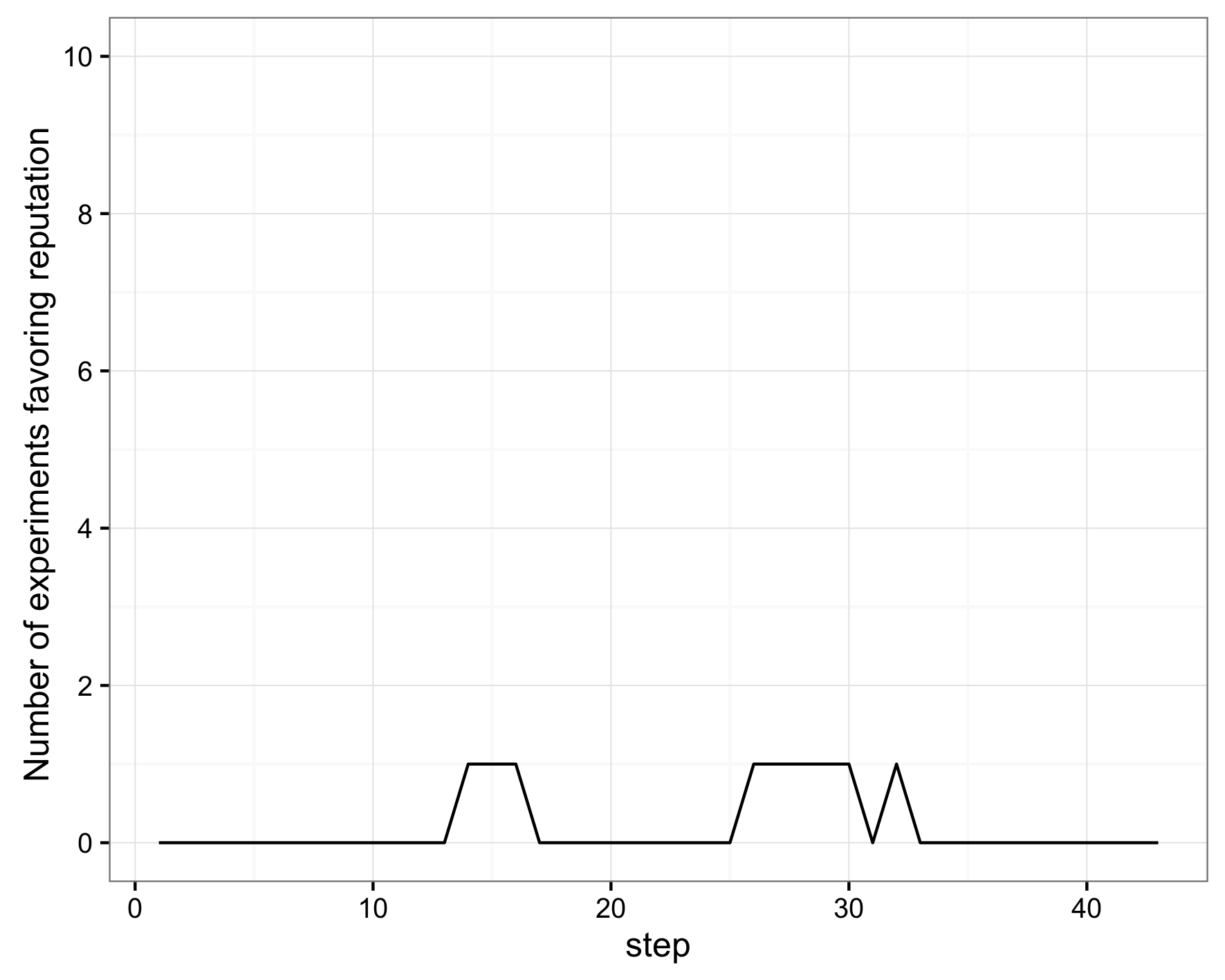

Supplement: Supplementary file 1 — Supplementary material 1 (zip 5147 KB) [file 11192_2018_2826_MOESM1_ESM.zip › ESM_1/v7-PR-zipf-40_z1548_agentsim_diff_sim.png]

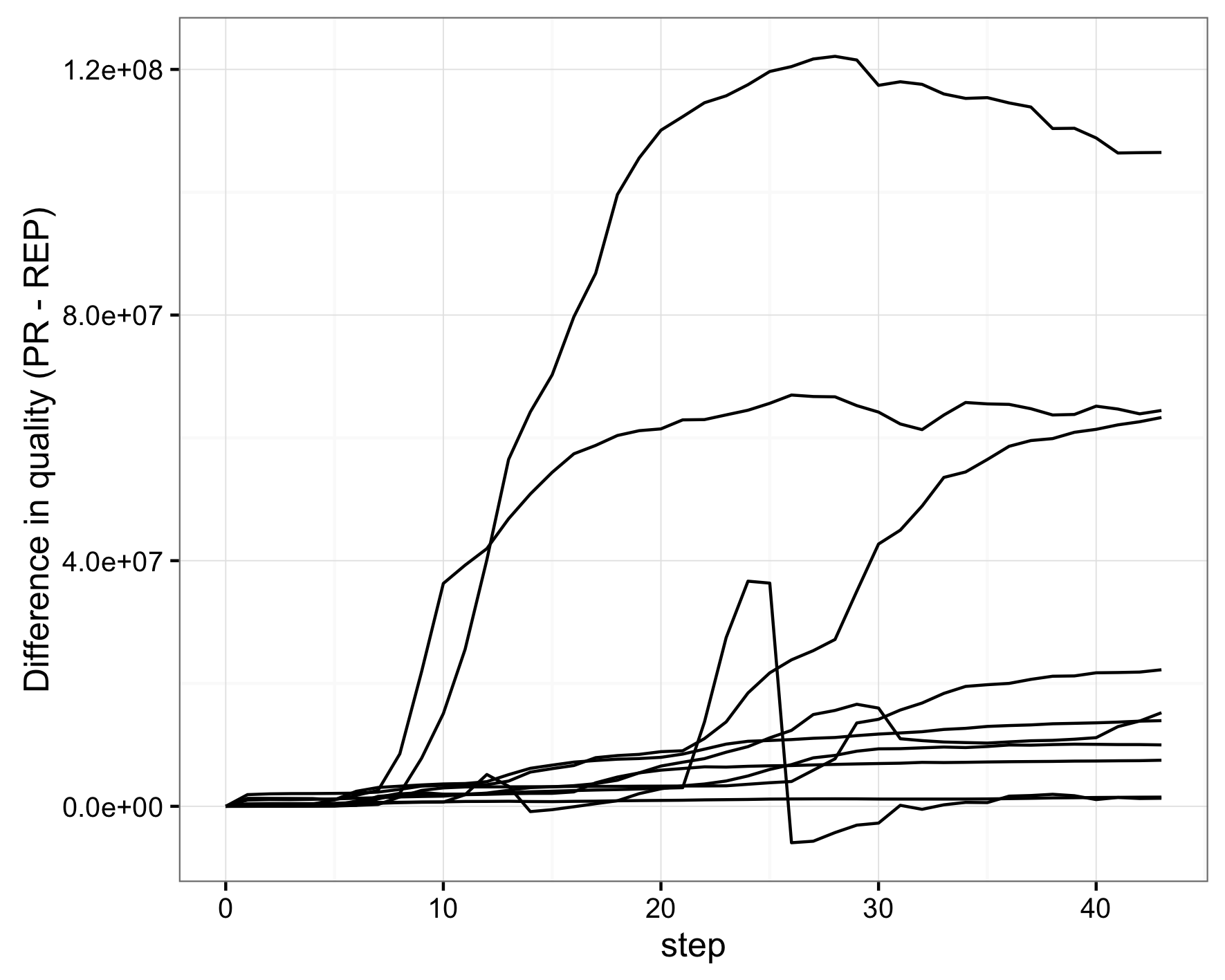

Supplement: Supplementary file 1 — Supplementary material 1 (zip 5147 KB) [file 11192_2018_2826_MOESM1_ESM.zip › ESM_1/v7-PR-zipf-40_z1548_agentsim_sim.png]

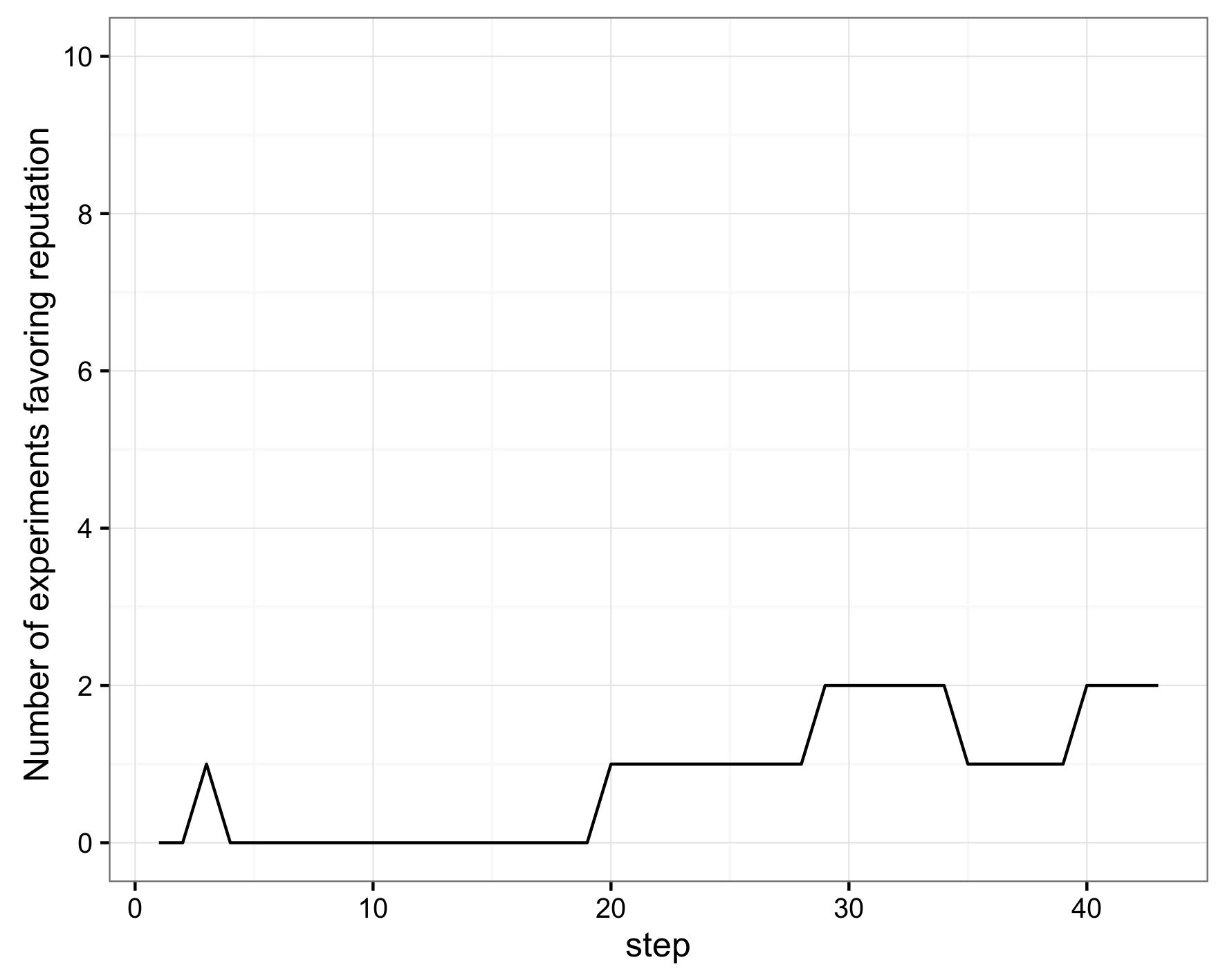

Supplement: Supplementary file 1 — Supplementary material 1 (zip 5147 KB) [file 11192_2018_2826_MOESM1_ESM.zip › ESM_1/v7-PR-zipf-40_z172_agentsim_diff_sim.png]

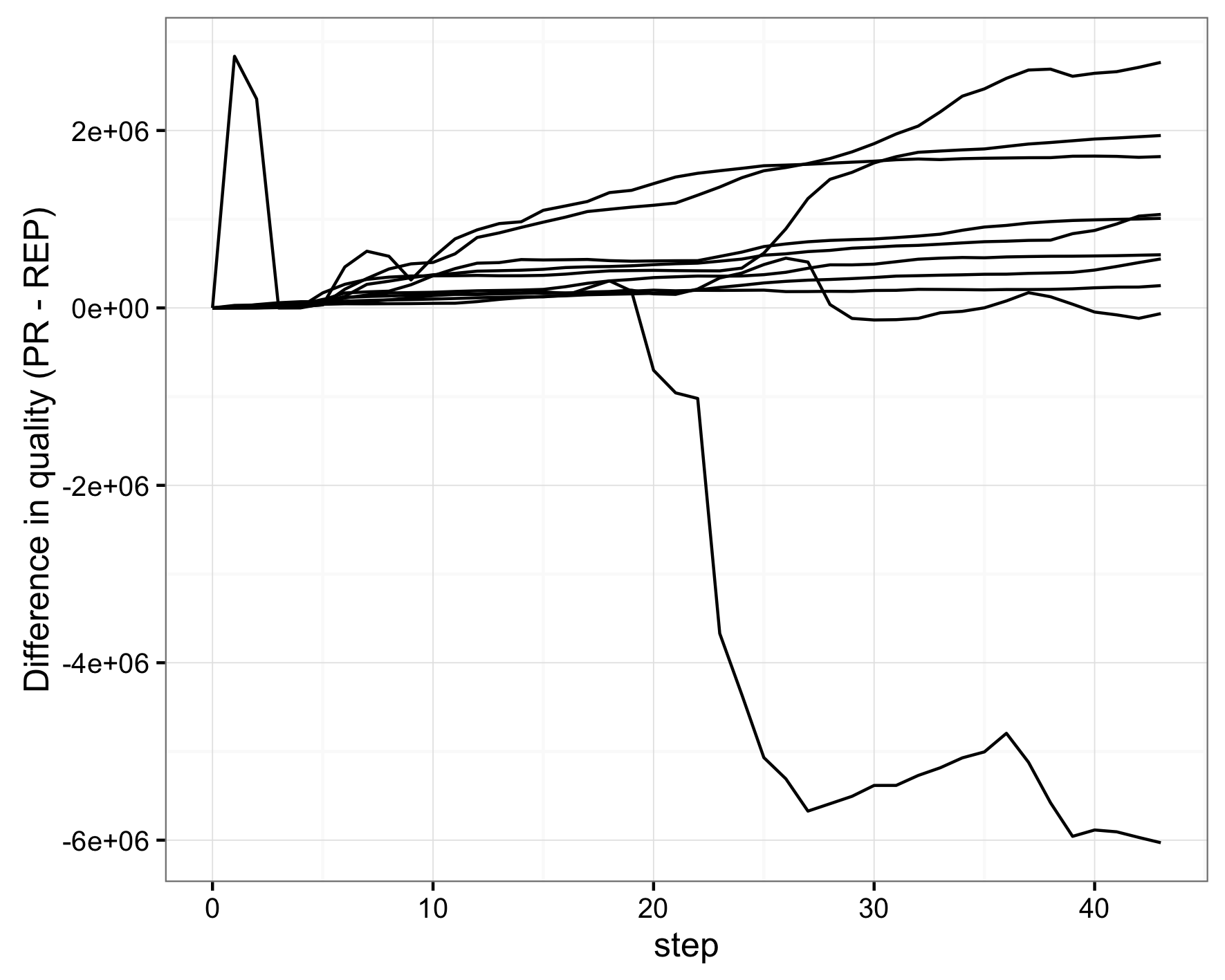

Supplement: Supplementary file 1 — Supplementary material 1 (zip 5147 KB) [file 11192_2018_2826_MOESM1_ESM.zip › ESM_1/v7-PR-zipf-40_z172_agentsim_sim.png]

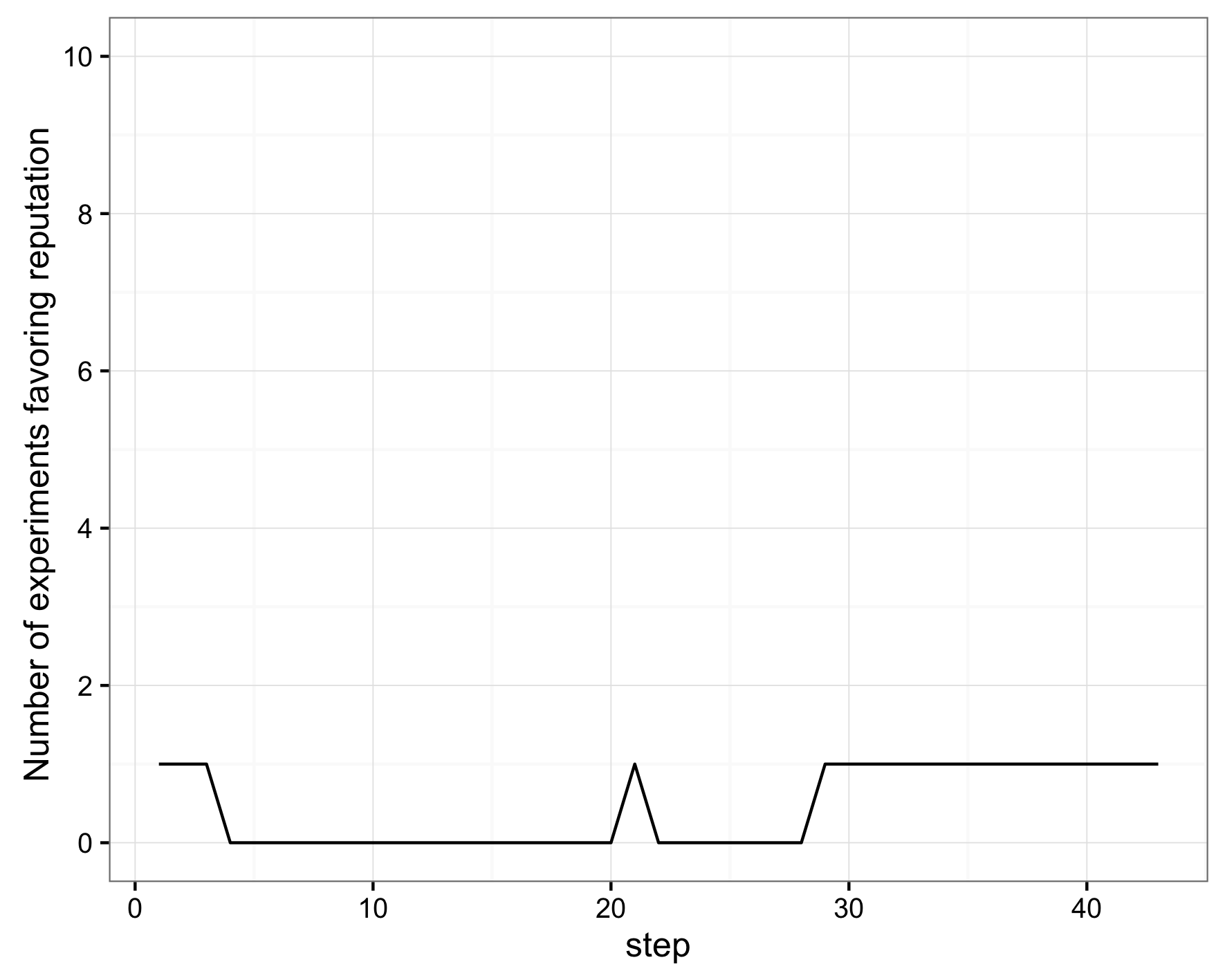

Supplement: Supplementary file 1 — Supplementary material 1 (zip 5147 KB) [file 11192_2018_2826_MOESM1_ESM.zip › ESM_1/v7-PR-zipf-40_z1892_agentsim_diff_sim.png]

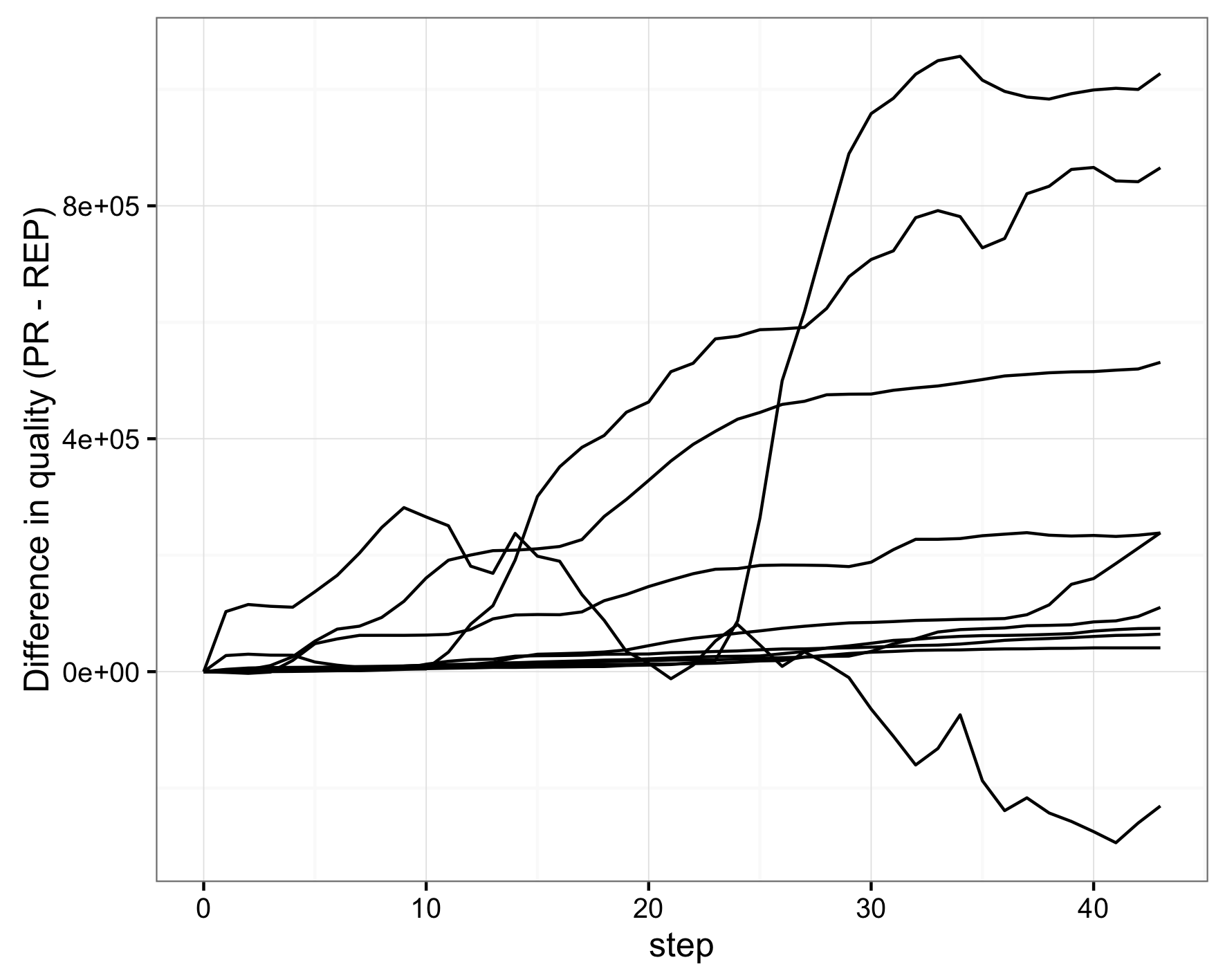

Supplement: Supplementary file 1 — Supplementary material 1 (zip 5147 KB) [file 11192_2018_2826_MOESM1_ESM.zip › ESM_1/v7-PR-zipf-40_z1892_agentsim_sim.png]

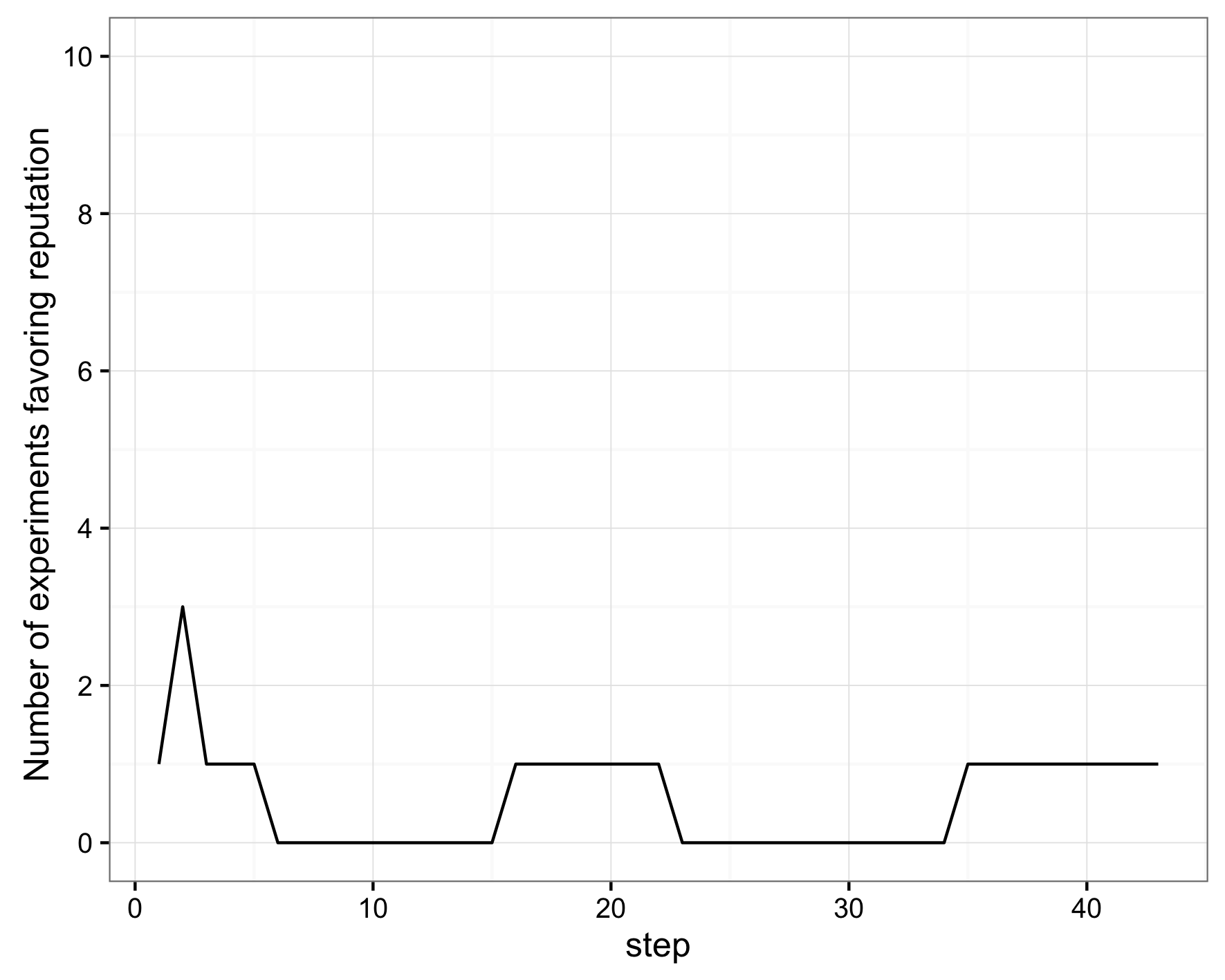

Supplement: Supplementary file 1 — Supplementary material 1 (zip 5147 KB) [file 11192_2018_2826_MOESM1_ESM.zip › ESM_1/v7-PR-zipf-42_p63_agentsim_diff_sim.png]

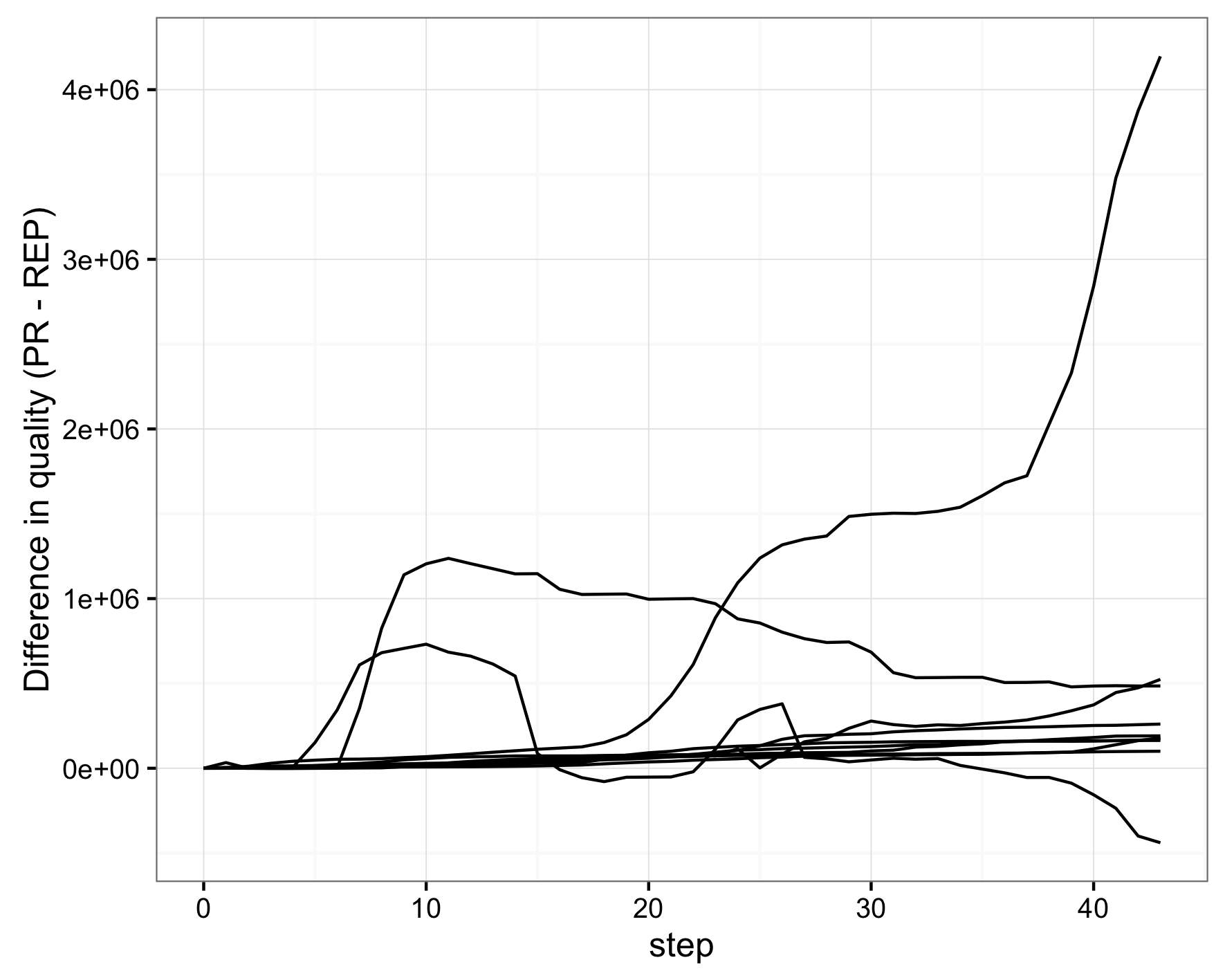

Supplement: Supplementary file 1 — Supplementary material 1 (zip 5147 KB) [file 11192_2018_2826_MOESM1_ESM.zip › ESM_1/v7-PR-zipf-42_p63_agentsim_sim.png]

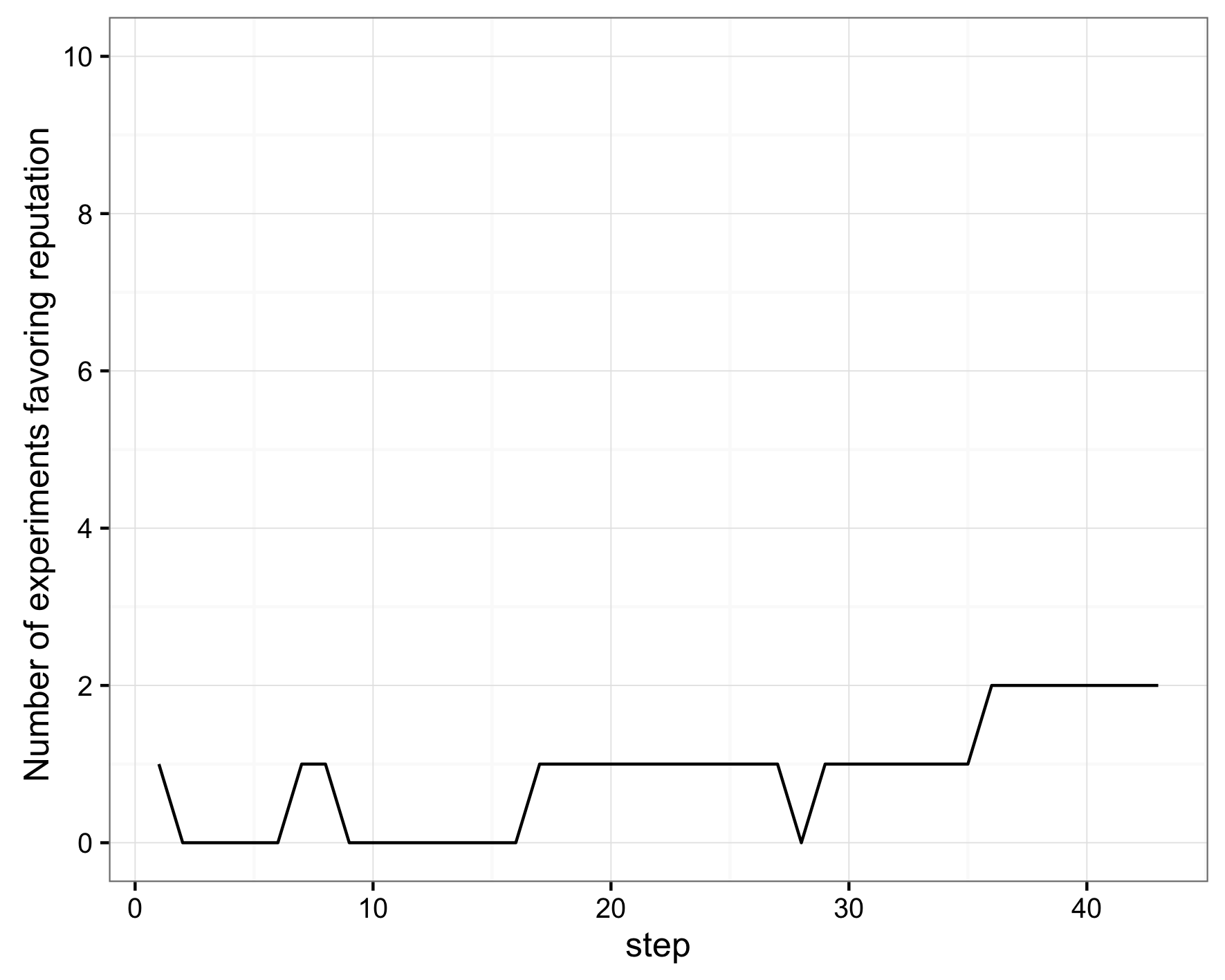

Supplement: Supplementary file 1 — Supplementary material 1 (zip 5147 KB) [file 11192_2018_2826_MOESM1_ESM.zip › ESM_1/v7-PR-zipf-42_p77_agentsim_diff_sim.png]

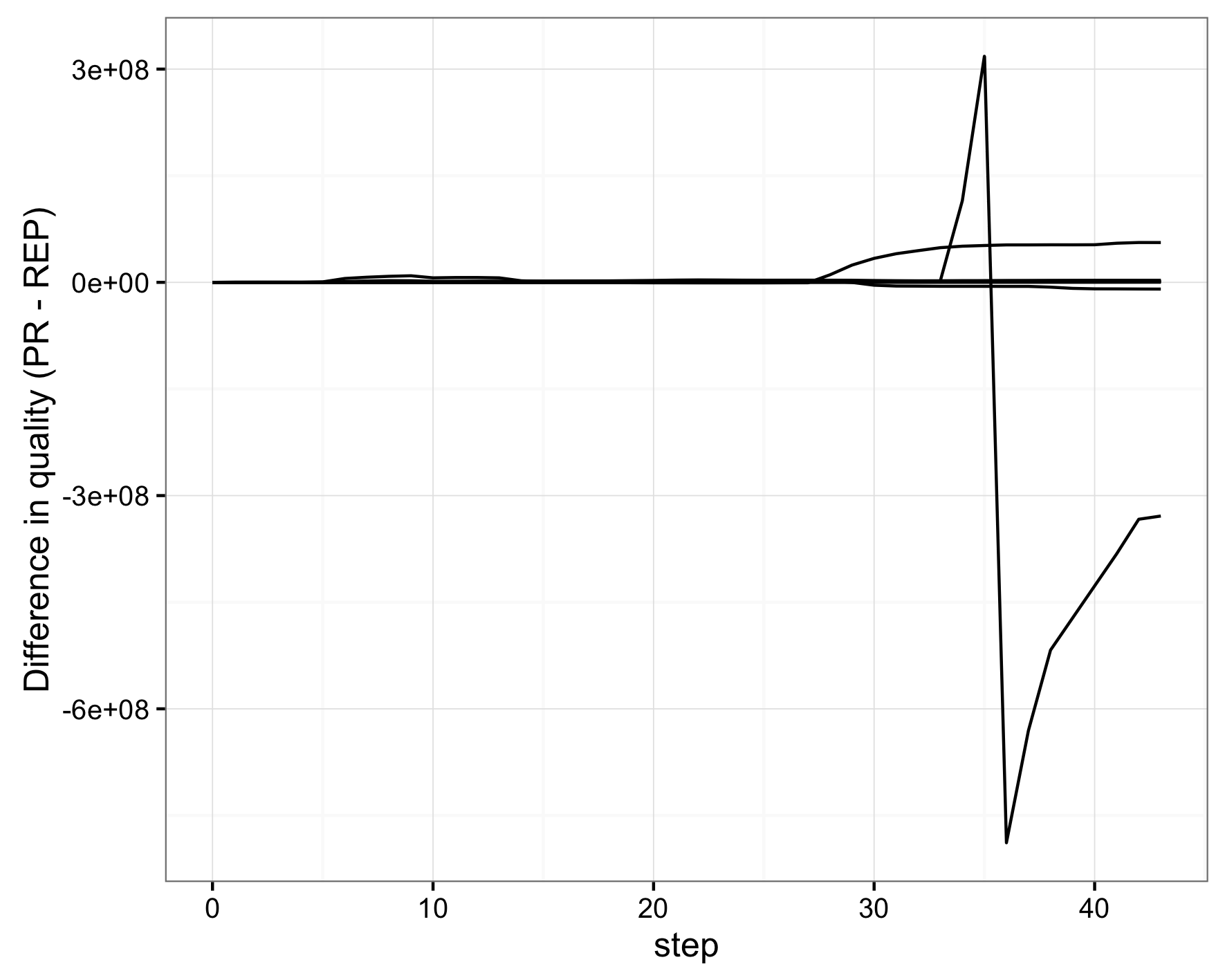

Supplement: Supplementary file 1 — Supplementary material 1 (zip 5147 KB) [file 11192_2018_2826_MOESM1_ESM.zip › ESM_1/v7-PR-zipf-42_p77_agentsim_sim.png]

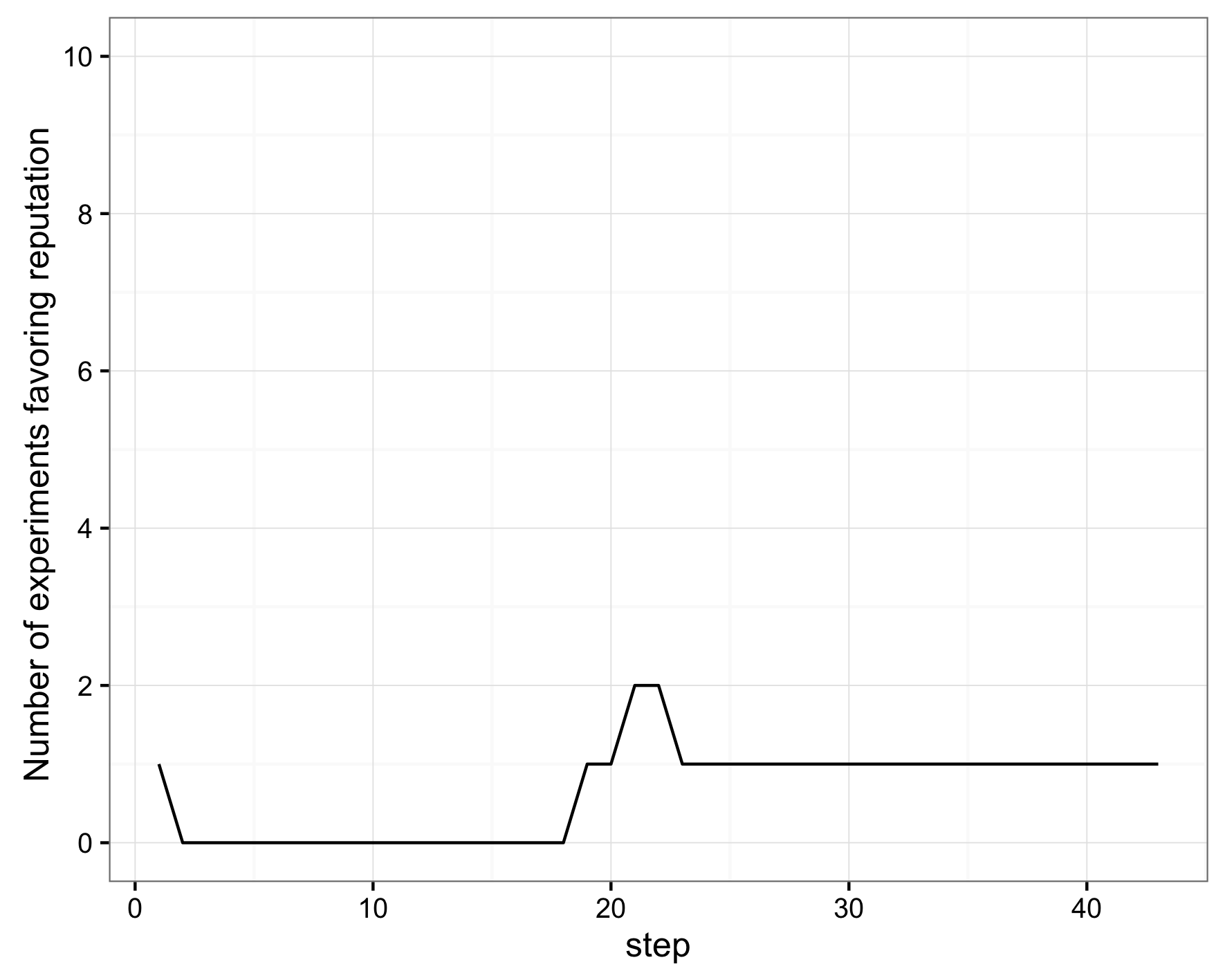

Supplement: Supplementary file 1 — Supplementary material 1 (zip 5147 KB) [file 11192_2018_2826_MOESM1_ESM.zip › ESM_1/v7-PR-zipf-42_p7_agentsim_diff_sim.png]

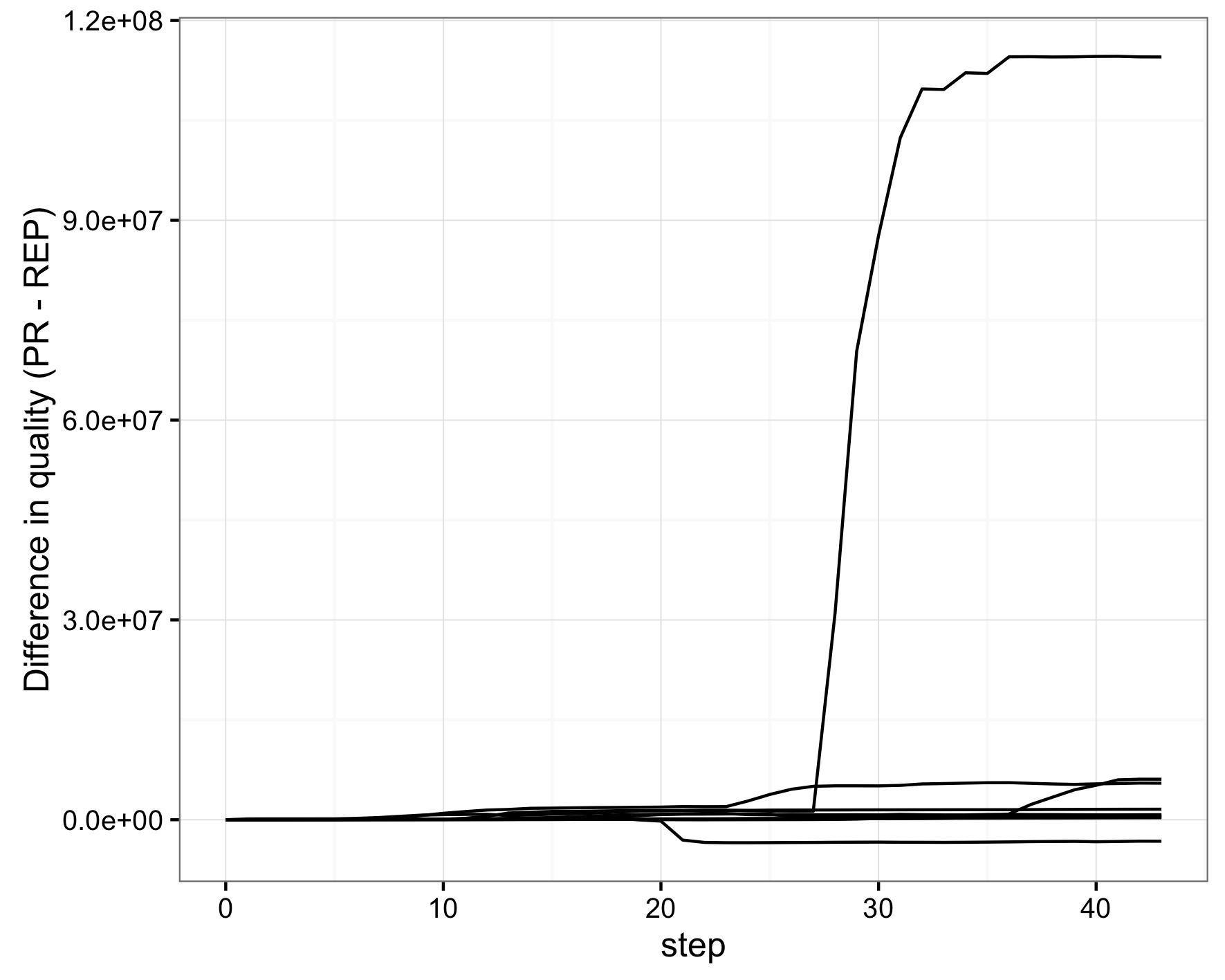

Supplement: Supplementary file 1 — Supplementary material 1 (zip 5147 KB) [file 11192_2018_2826_MOESM1_ESM.zip › ESM_1/v7-PR-zipf-42_p7_agentsim_sim.png]

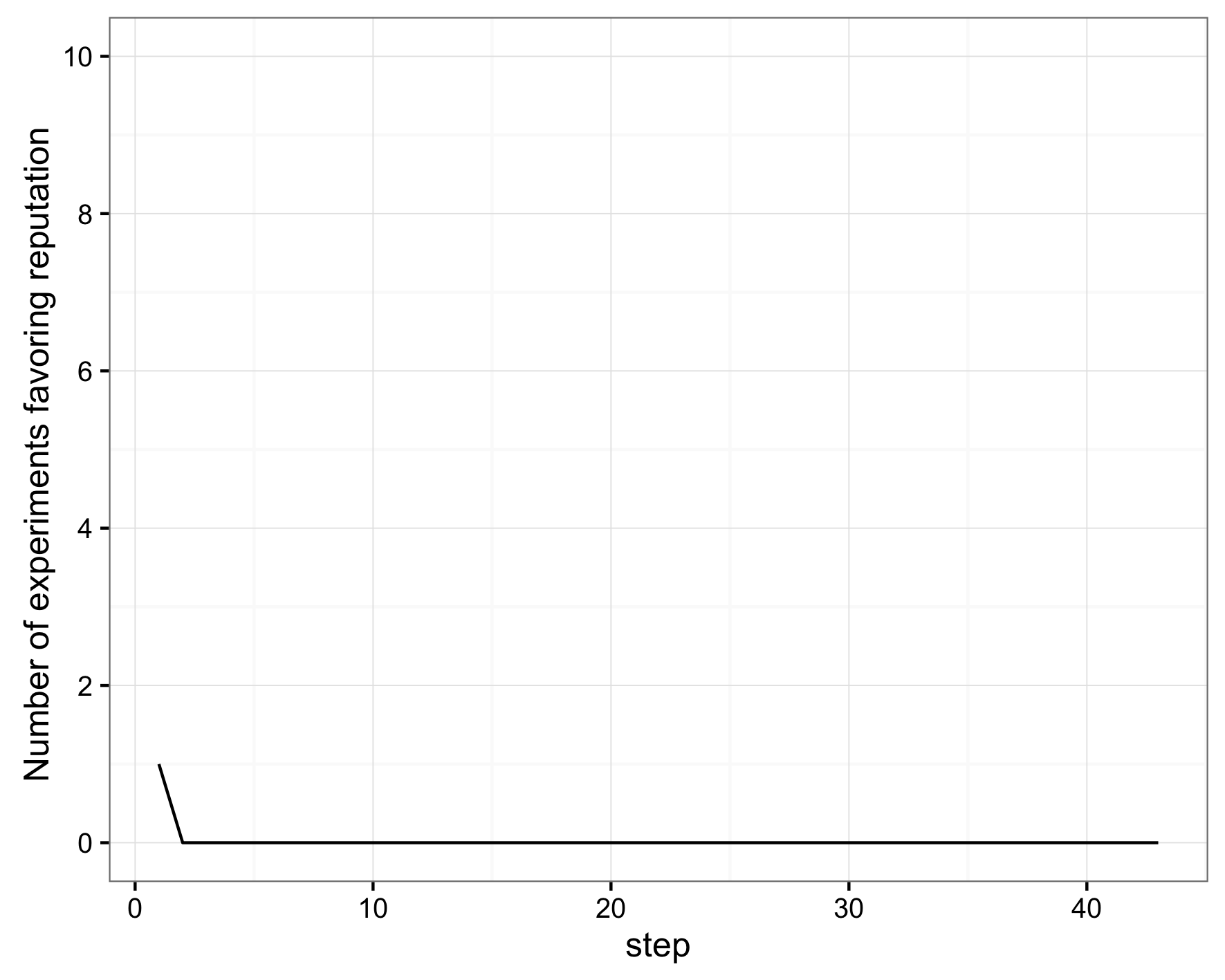

Supplement: Supplementary file 1 — Supplementary material 1 (zip 5147 KB) [file 11192_2018_2826_MOESM1_ESM.zip › ESM_1/v7-PR-zipf-44_j35_diff_sim.png]

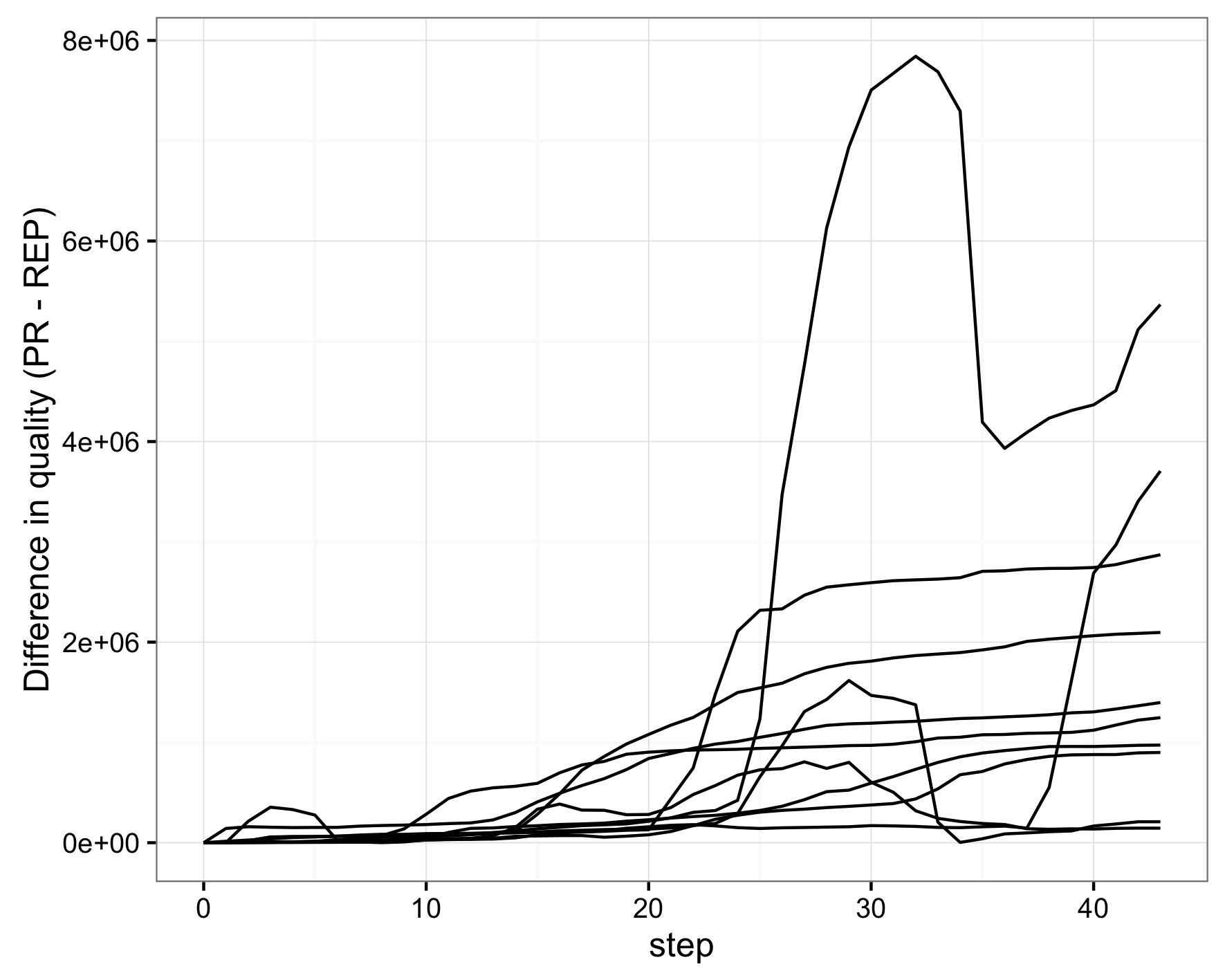

Supplement: Supplementary file 1 — Supplementary material 1 (zip 5147 KB) [file 11192_2018_2826_MOESM1_ESM.zip › ESM_1/v7-PR-zipf-44_j35_sim.png]

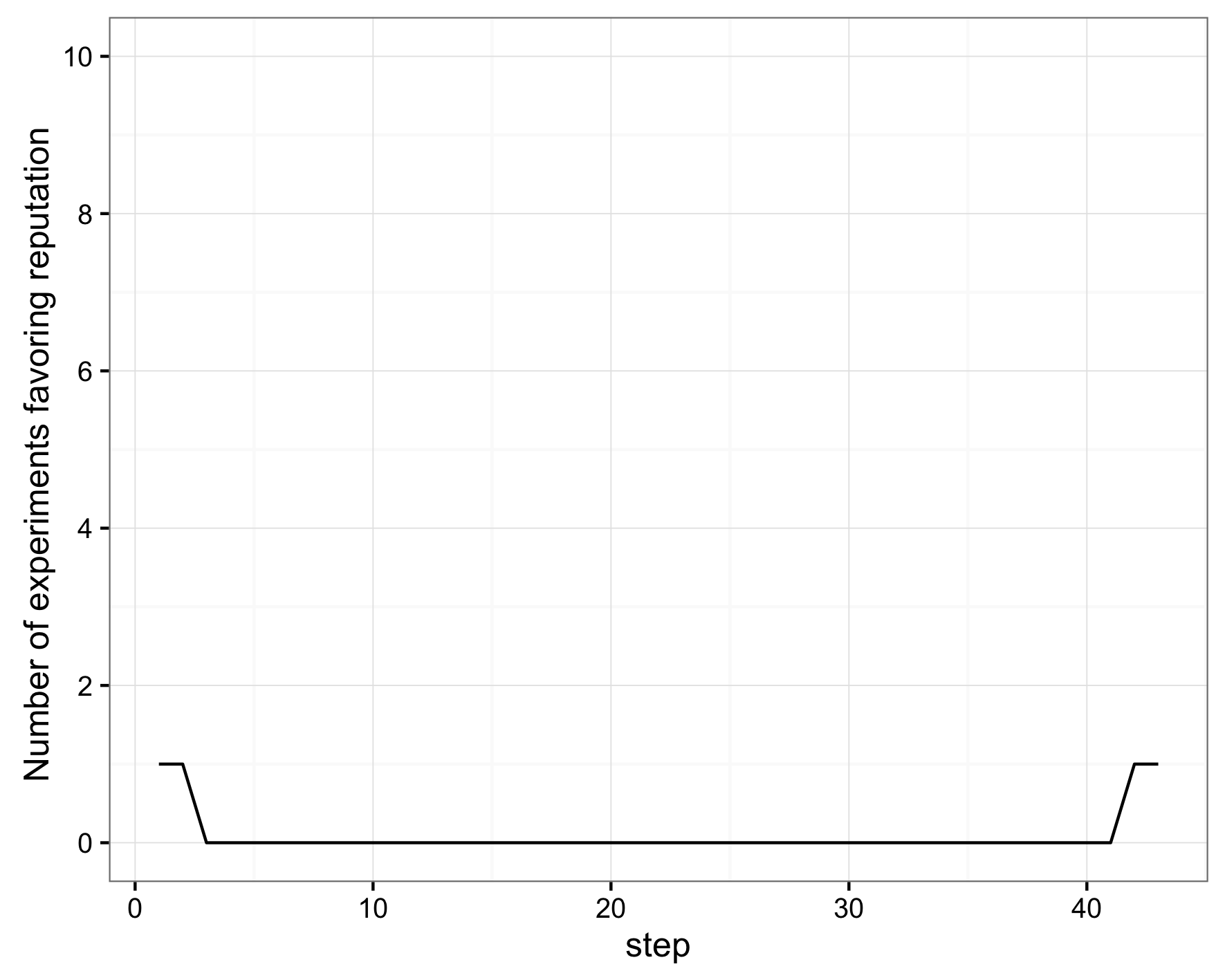

Supplement: Supplementary file 1 — Supplementary material 1 (zip 5147 KB) [file 11192_2018_2826_MOESM1_ESM.zip › ESM_1/v7-PR-zipf-44_j37_diff_sim.png]

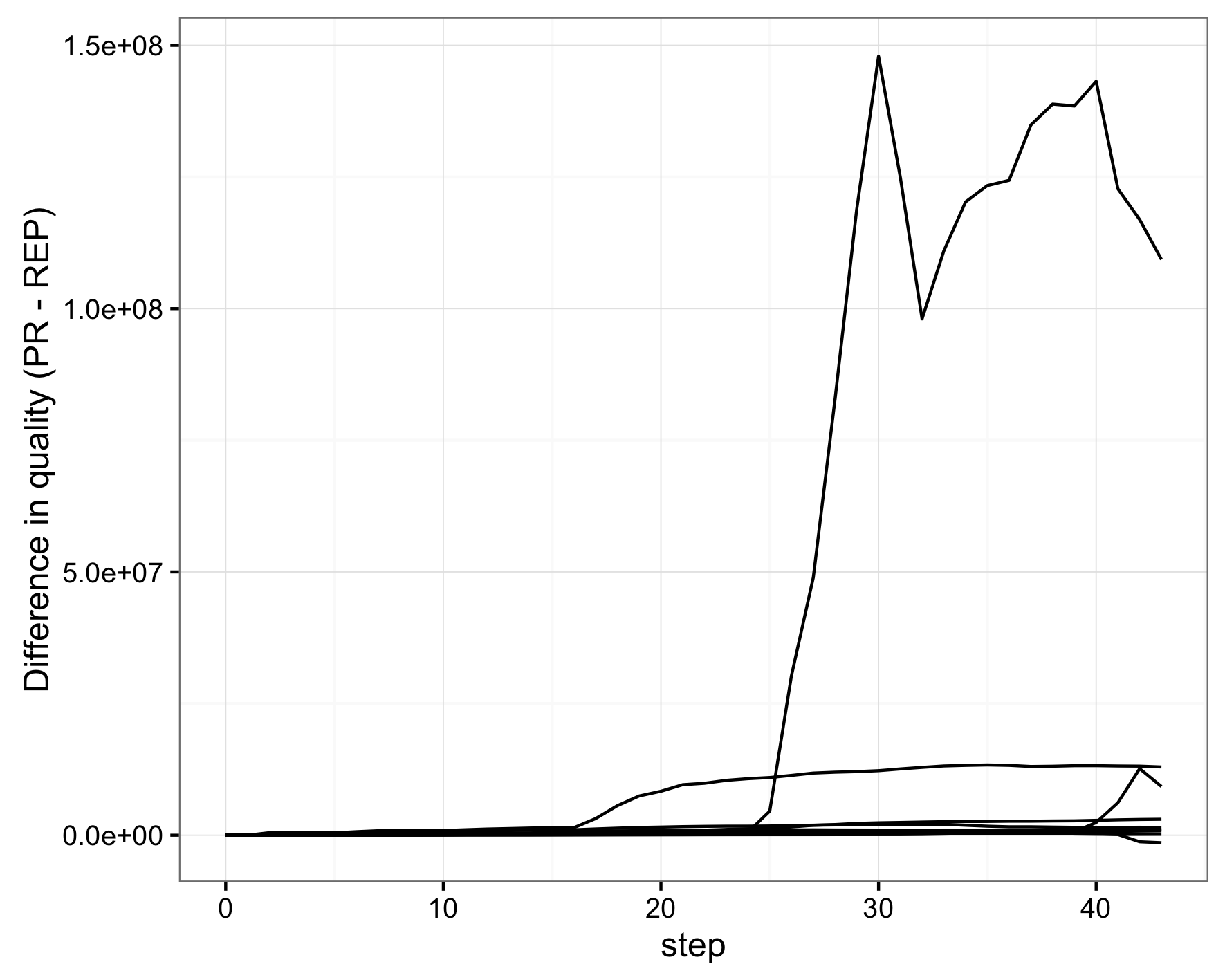

Supplement: Supplementary file 1 — Supplementary material 1 (zip 5147 KB) [file 11192_2018_2826_MOESM1_ESM.zip › ESM_1/v7-PR-zipf-44_j37_sim.png]
